# Biosynthesis of silver nanoparticles by *Talaromyces funiculosus* for therapeutic applications and safety evaluation

**DOI:** 10.1038/s41598-025-95899-7

**Published:** 2025-04-21

**Authors:** Bahig A. El deeb, Gerges G. Faheem, Mahmoud S. Bakhit

**Affiliations:** 1 Department of Botany and Microbiology, Faculty of Science, Sohag University, Sohag, 82524 Egypt; 2 Higher Technological Institute of Applied Health Science in Sohag, Ministry of Higher Education, Cairo, Egypt

**Keywords:** *Talaromyces funiculosus*, Extracellular biosynthesis, Characterization and optimization, Antioxidant, Anti-inflammatory, Biological techniques, Biotechnology, Microbiology

## Abstract

The study investigated the capacity of the endophytic fungus *Talaromyces funiculosus* to biosynthesize extracellular AgNPs and assess their safety. The fungus was identified through morphological and phylogenetic analyses. The biosynthesized AgNPs were spherical crystalline, stable (6 months), and mono-dispersed (PDI: 0.007), exhibiting SPR at 422.5 nm, average diameter of 34.32 nm, and Zeta potential of -18.41 mV. The optimal biosynthesis conditions are 1 mM AgNO_3_, 5 g biomass, pH 5.5, and a reaction temperature of 60 °C. *Escherichia coli* (bacterial strains) and *Candida tropicalis* (yeast strains) exhibited the highest susceptibility with inhibition zones of 26.3 mm and 22.3 mm, respectively, at 50 µg/mL of AgNPs, and MICs of 3.7 µg/mL and 6.3 µg/mL, respectively. AgNPs exhibited cytotoxicity with IC_50_ values of 48.11 ppm for HEK-293 and 35.88 ppm for Hep-G2 cells, showing selective toxicity toward cancer cells. They demonstrated antioxidant activity by increasing GSH (10.29 to 14.76 mmol/g) and reducing MDA (40.57 to 26.28 nmol/ml) at 48.11 ppm. AgNPs also enhanced IL-10 production (96.47 to 177.0 pg/mL) and reduced TNF-α levels (55.77 to 41.06 pg/mL), indicating their anti-inflammatory properties. These results support the safe use of low-dose AgNPs, however, further studies are needed to evaluate AgNPs for clinical uses.

**Supplementary Information:**

The online version contains supplementary material available at 10.1038/s41598-025-95899-7.

Silver nanoparticles (AgNPs) continue gaining significant interest in Nanotechnology, particularly in biomedicine, due to their intriguing properties and potential applications^1–3^. They have various applications in the fields of antimicrobial, anticoagulant, anticancer, and drug delivery^4^. Their significance stems from their catalytic activity, chemical stability, and thermal properties^5^. Silver nanoparticles have been employed in biomedical applications to prevent infections and promote wound healing^6^. They have demonstrated strong antimicrobial activity against a variety of microorganisms^7,8^. The high surface-to-volume ratio of AgNPs is a critical factor contributing to their antimicrobial activity^9^. Eco-friendly approach for the green synthesis of AgNPs, which involves natural reducing and capping agents, has gained significant interest as an alternative to chemical synthesis protocols that are resource-intensive and potentially harmful^10^. The synthesis of nanoparticles can be intracellular or extracellular according to the location where nanoparticles are formed^11^.

Fungi have shown greater efficiency in the synthesis of AgNPs compared to plants and bacteria^12–14^. Fungus cultures have the advantage that they can provide effective biomass production, no additional steps are required to extract the filtrate, their mycelia offer a large surface area for interactions, and exhibits higher resistance toward agitations and pressures^15^. Fungi secrete substantial quantities of enzymes and proteins, significantly enhancing productivity and ensuring protein coating, high stability, and prevention of nanoparticle agglomeration^16^. The extracellular biosynthesis of AgNPs by endophytic fungi could make downstream processing much easier than the intracellular biosynthesis^17^. Recent studies have focused on using fungal cellular filtrate to synthesize AgNPs, with successful results^18^. Scaling up microbial-derived nanoparticle production requires optimizing growth conditions through various techniques to achieve favorable large-scale synthesis^19^. Enhancing the production of biosynthesized AgNPs by endophytic fungi requires optimization of culture conditions and key physical parameters, such as pH and temperature^20^.

AgNPs are widely recognized as highly effective antimicrobial agents that have minimal toxic effects on healthy mammalian cells^21^. The daily intake of silver from natural sources in food and water consumed by humans ranges from approximately 0.4 to 30 µg^22^. The size and shape of nanoparticles significantly influence the toxicity of AgNPs, with 10 nm AgNPs being more toxic than 50, 100, and 200 nm particles^23^. The toxicity of AgNPs is mainly attributed to the release of silver ions, with spherical-shaped AgNPs showing less toxicity compared to other nanoparticle shapes^24^. AgNPs induce oxidative damage to cell membranes and organelles, including the nucleus, mitochondria, and lysosomes, consequently triggering inflammatory responses^25^.

Silver nanoparticles have garnered significant attention for their diverse applications across healthcare, environmental management, agriculture, and industry, owing to their antimicrobial, antiviral, anti-inflammatory, and antioxidant properties^26^. They enhance plant growth by improving seed germination, increasing crop yields, and serving as non-toxic alternatives to chemical pesticides and fertilizers^27^. They are used in food storage, textile coatings, and water purification, where their antimicrobial properties enhance food shelf life, reduce bacterial spread in textiles, and eliminate pathogens in water^28^. AgNPs are integrated into medical devices like wound dressings, catheters, and bone cement, where they prevent infections and promote healing. They have recently gained attention as potent anticancer agents due to their ability to induce apoptosis in various human cancer cell lines^29,30^. Despite their broad potential, concerns regarding the toxicity of AgNPs to humans and the environment persist^31^. AgNPs can accumulate in organs such as the liver, kidneys, brain, and lungs, potentially causing long-term health issues, including lung inflammation, neurodegeneration, and immune suppression, with prolonged exposure may intensify these risks^32^. Additionally, the release of AgNPs and silver ions into the environment may disrupt microbial communities^33^. Continued research is necessary to understand their safety, environmental impact, and long-term health effects.

*Euphorbia hirta* L. (Euphorbiaceae) commonly known as milkweed or asthma plant is widely distributed worldwide^34^. This plant is extensively used in traditional medicine, particularly in China and India, due to its antibacterial and antifungal properties^35^. Several endophytic fungi were isolated from it, including *Alternaria arborescens*, *Cladosporium cladosporioides*, and *Rhizopus oryzae*^36^, as well as *Nigrospora sphaerica*^37^ and *Fusarium nectrioides*^38^.

*Talaromyces funiculosus* (Aspergillaceae) is of significant mycological interest due to its diverse applications and ability to adapt to various habitats, including plants, decaying organic matter, and soil^39^. *Talaromyces funiculosus* produces a wide range of secondary metabolites, including phytohormones, a complex of hydrolytic enzymes, and phenolic substances^40^. Recent studies have explored the use of non-pathogenic strains of *T. funiculosus* for producing sweet flavor compounds^41^, cellulose, xylanase^42^, chitinase enzyme^43^, and flavonoid compounds^44^.

This study explores the extracellular biosynthesis of AgNPs by a non-pathogenic strain of *T. funiculosus*. The synthesis of AgNPs was optimized and characterized, followed by an evaluation of their antimicrobial properties against various pathogenic bacteria and yeast strains. The anticancer and cytotoxicity were assessed on Hep-G2 cancer cells and HEK-293 normal cells. Notably, this study provides a comprehensive evaluation of the safety of AgNPs by examining a wide array of biomarkers, including antioxidant biomarkers (total antioxidant capacity (TAC), catalase (CAT), reduced glutathione (GSH), and superoxide dismutase (SOD)), oxidative stress indicators (malondialdehyde (MDA) and nitric oxide (NO)), inflammatory cytokines (tumor necrosis factor alpha (TNF-α), human interleukin 6 (IL-6), and human interleukin 1beta (IL-1β)), and the anti-inflammatory cytokine IL-10. This integrated approach offers a novel insight into the potential therapeutic and safety profile of AgNPs.

## Results and discussion

### The isolated endophytic fungi from *E. hirta*

Three fungal species, *Nigrospora gorlenkoana*, *Talaromyces funiculosus*, and *Verticillium* sp., were isolated from healthy leaves of *E. hirta*. Among these, *T. funiculosus* was selected for further investigation due to its rapid growth and higher biomass production in a shorter time compared to the other species. It was subsequently identified through morphological and phylogenetic analyses.

### Identification of the selected endophytic fungus

The taxonomical position of *Talaromyces funiculosus* (SUMCC 22011) was confirmed based on morphological and molecular studies in this article.

### Phylogenetic analysis

**Fig. 1:**
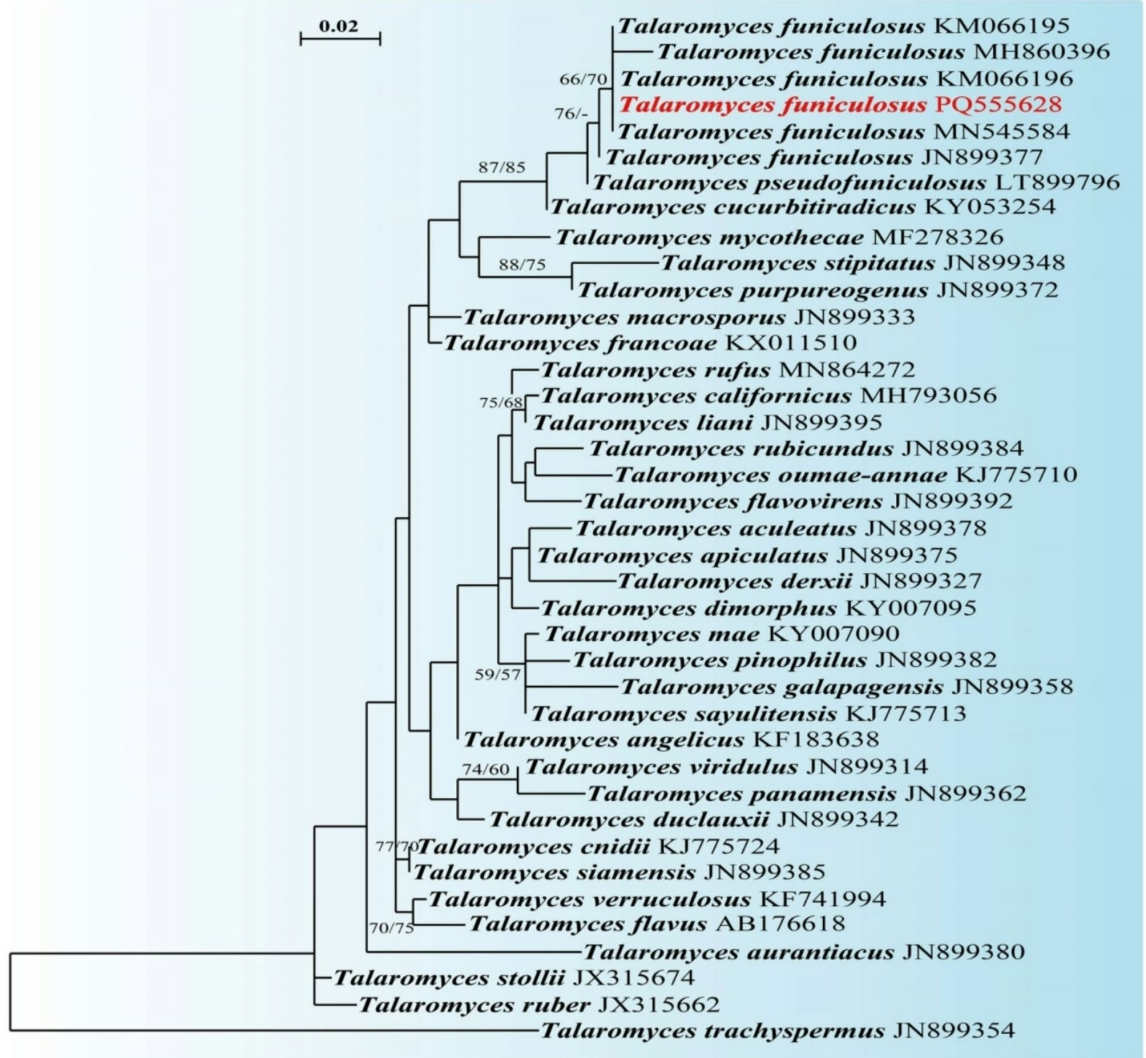
Phylogram generated from ML analysis (RAxML) based on ITS sequence dataset for *Talaromyces funiculosus* (SUMCC 22011) along with other species of *Talaromyces* section *Talaromyces*. Bootstrap support on the nodes represents ML and MP ≥ 50%. The sequence of our new isolate is in red.

The ITS dataset consisted of 38 taxa belong to the genus *Talaromyces* section *Talaromyces* and *Talaromyces trachyspermus* as outgroup. The RAxML analysis of the ITS dataset yielded the best-scoring tree (Fig. 1) with a final ML optimization likelihood value of -1467.409581. The matrix had 104 distinct patterns with 6.27% undetermined characters or gaps. Estimated base frequencies were found to be A = 0.189129, C = 0.283983, G = 0.315569, T = 0.211320; substitution rates, AC = 1.875428, AG = 2.307920, AT = 1.865142, CG = 0.000100, CT = 7.384141, GT = 1.0. Our phylogenetic analysis placed the new isolate of *Talaromyces funiculosus* (SUMCC 22011) within *Talaromyces* section *Talaromyces* and clusters with other strains of *T*. *funiculosus* including the type strain of *T*. *funiculosus* CBS 272.86.

### Taxonomy

**Fig. 2:**
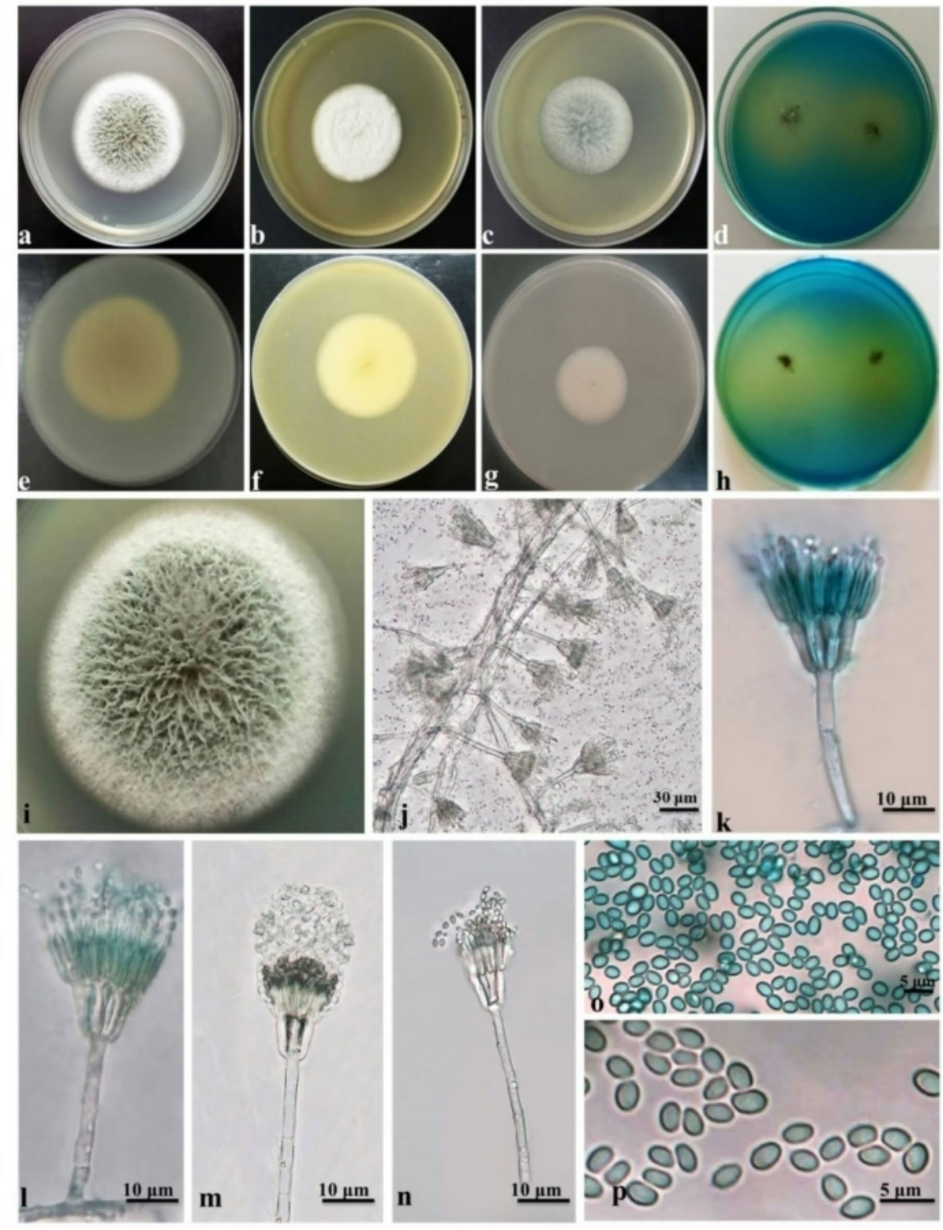
*Talaromyces funiculosus* (SUMCC 22011): (**a**–**d**) colonies on CYA, MEA, PDA and CREA, (**e**–**h**) the reverse for the same media, respectively, (**i**) colony texture on MEA after 5 days incubation, (**j**–**n**) conidiophores, (**o**,**p**) conidia.

***Talaromyces funiculosus*** (Thom) Samson et al., *Stud. Mycol.* 71: 176. 2011^45^. (Fig. 2)

**GenBank accession numbers**, ITS: PQ555628.

**Colony diameter** after 7 d (mm) at 25 °C: CYA 40–45; MEA 32–40; PDA 35–40; CREA 8–12. The diameter at 30 °C: CYA 42–48; MEA 35–40; PDA 35–45; CREA 12–15.

**Colony characters** after 7 d (mm) at 25 °C: On CYA, Colonies slightly raised at center, margins low, plane, entire; mycelia white; texture floccose; sporulation absent to moderately dense, conidia en masse greyish green to dull green. On MEA, colonies moderately deep, plane; margins low, plane, entire; mycelia white; texture funiculose; sporulation sparse to dense, conidia en masse greyish green to dull green, soluble pigments absent; exudates absent; reverse pale yellow to yellow. On PDA, colonies moderately deep, plane; margins low, plane, entire; mycelia white; texture funiculose; sporulation dense, conidia en masse greyish green to dull green, reverse pale yellow to yellow. The fungus was showed strong acid production on CREA medium.

**Micromorphology**: on MEA after 7 days of growth at 25 °C: Conidiophores biverticillate with a minor proportion having sub-terminal branches; stipes smooth walled, 25–65 × 2–3 μm; branches 15–25 μm; metulae 2–6, 8–13 × 1–3 μm, appressed to divergent, phialides acerose, 3–5 per metulae, 7–12 × 1–2 μm; conidia smooth, ellipsoidal, 1–3 × 1–2 μm. Chlamydospores are absent and ascomata not observed.

**Materials examined**: Egypt, Sohag Governorate, Wadi Bir-EL-Ain (26°38’36.8"N 31°50’13.5"E), from healthy leaves of *Euphorbia hirta* (Euphorbiaceae), Feb. 2022, coll. G. G. Faheem, The culture is deposited in Sohag University microbial culture collection, Egypt (SUMCC 22011).

**Notes**: *Talaromyces funiculosus* was distinguished by the formation of funiculose colonies and strong acid production in the CREA medium. Morphologically, our strain was similar to *T*. *funiculosus*, as described by Yilmaz et al.^39^ and Liu et al.^46^. This species is commonly isolated from soil, indoor environments, and food products. It is also an endophytic fungus associated with a variety of plant hosts, including *Cotyledon orbiculata*, *Melianthus comosus*, *Psychotria zombamontana*, and *Oxycoccus palustris*^39,42,47^.

### Biosynthesis and characterization of AgNPs

The isolated endophytic fungi were subjected to a preliminary investigation to test their ability to biosynthesis of AgNPs. The biosynthesis of AgNPs was carried out by react the obtained filtrate from 10 g of fungal biomass with silver nitrate (AgNO_3_) to reach a final concentration of 1 mM silver ions at pH 7.0 and 28 °C, under dark conditions. The reaction was performed in triplicate. The results demonstrated that *T. funiculosus* was capable of synthesizing AgNPs, whereas the other two fungi (*N. gorlenkoana* and *Verticillium* sp.) did not show ability to biosynthesize AgNPs under the same conditions.

**Fig. 3:**
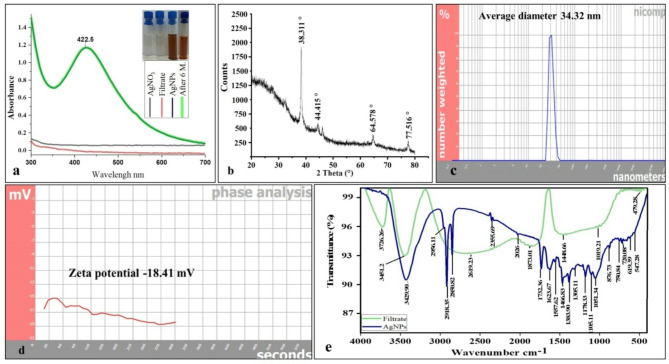
Characterization of the biosynthesized AgNPs by *T. funiculosus*: (**a**) UV-visible absorption spectra, (**b**) XRD Pattern, (**c**) DLS measurements, (**d**) Zeta potential analysis, (**e**) FTIR analysis.

The biosynthesis of AgNPs by *T. funiculosus* was indicated by a noticeable color change in the reaction mixture from pale yellow to brown. The color change signifies the reduction of silver ions to AgNPs^26^. This was further confirmed by UV-visible spectroscopy, which revealed a distinct surface Plasmon resonance (SPR) peak of AgNPs at 422.5 nm (Fig. 3a), consistent with the typical range of 400–500 nm^48^. No color changes or significant shifts in the UV-visible spectra were observed in the control samples. Similarly, other endophytic fungi have also demonstrated the ability to synthesize AgNPs: *Exserohilum rostratum* exhibiting a SPR at 420 nm^49^, *Penicillium brasilianum* at 420 nm^29^, *Exserohilum rostrata* at 425 nm^50^, and *Phomopsis helianthi* at 422 nm^51^.

The biosynthesized AgNPs exhibited excellent stability over six months at room temperature, with no precipitation or changes in characteristics, as evidenced by the consistent SPR peak at 422.5 nm, indicating the preservation of their structural integrity (Fig. 3a). This stability is crucial for their potential therapeutic applications, as it ensures that AgNPs will not aggregate over time, which could compromise their efficacy and safety^52^. Similar findings were reported by Hao et al.^53^ and Pham et al.^54^, where AgNPs maintained their stability over six months with no shift in the SPR peak.

The synthesized AgNPs exhibited a crystalline structure, as confirmed by XRD analysis. Examination of the XRD pattern (Fig. 3b) demonstrated the presence of a face-centered cubic (FCC) lattice of silver, with diffraction peaks observed at 38.311°, 44.415°, 64.78°, and 77.516° corresponding to the (111), (200), (220), and (311) planes, respectively (Pattern: COD 9011607).

DLS analysis of synthesized AgNPs displayed a size range of 30 to 49 nm, with an average diameter of about 34.32 nm (Fig. 3c). Zeta potential of synthesized AgNPs was found to be -18.41 mV (Fig. 3d). These values indicate that the colloids demonstrate moderate stability, likely attributed to the presence of fungal proteins serving as a capping agent with a negative charge on the surface of the synthesized AgNPs^29^. This leads to robust repulsive forces among the particles, preventing agglomeration^55^. The AgNPs with Zeta potential above ± 30 mV are considered highly stable^56^.

FTIR analysis of the synthesized AgNPs was conducted to determine the factors involved in their production and stabilization. The FTIR spectra of AgNPs and fungal filtrate (Fig. 3e) exhibited common peaks, such as the stretching of –NH and –OH at approximately 3400 cm^− 1^. Significant characteristic peaks representing the biosynthesized AgNPs were also observed, indicating the formation of new bonds that was absent in the fungal filtrate. For instance, peaks at 2956, 2918, and 2850 cm^− 1^ represented C–H stretching of alkanes^56^. Peaks at 2355 and 1732 cm^− 1^ corresponded to –NH2 and C = O, respectively. The band at 2026 cm^− 1^ corresponded to N = C = S or N = C = N bond, and peaks at 1623, 1557, and 1466 cm^− 1^ were assigned to amide I, amide II, and C = C bonds, respectively^57^. Furthermore, bands observed at 1383, 1305, and 1178 cm^− 1^ were associated with C–N stretching vibrations of amine, while peaks at 1105, 1051, and 720 cm^− 1^ corresponded to C–O, –C–S–S stretching of disulfide linkage. The close similarity of these bands to those reported for native proteins indicated the role of the fungal extracellular protein as a reducing, capping, and stabilizing agent for the AgNPs^58^.

**Fig. 4:**
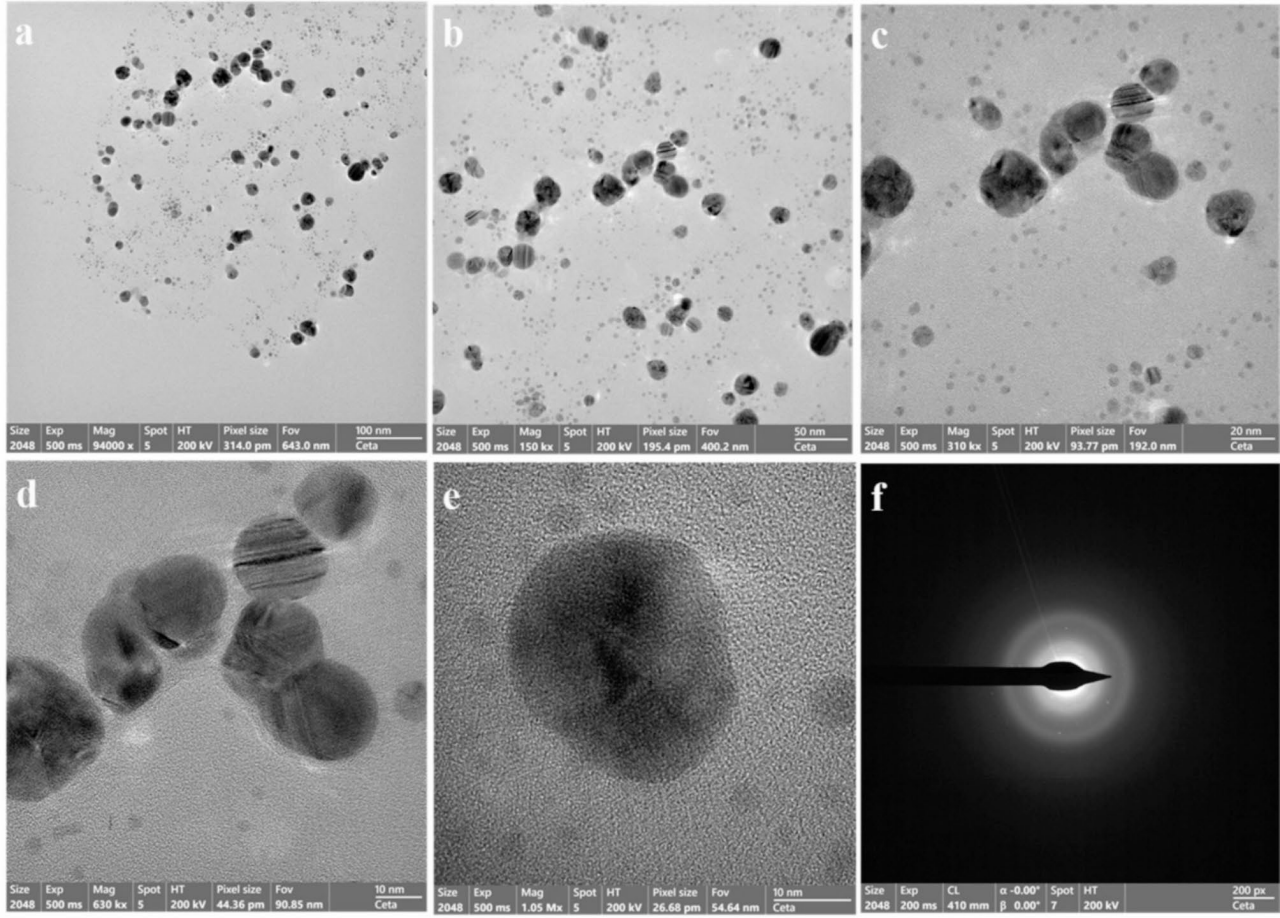
TEM images of AgNPs: (**a**–**e**) AgNPs images at different scale bar, (**f**) SAED pattern.

The synthesized AgNPs were characterized by TEM analysis to study its morphology. The TEM images were captured at magnifications of 94000X to 1.05 MX, as shown in Fig. 4a-e. The TEM micrograph reveals spherical nanoparticles with well-defined boundaries. The particles appear to be discrete and non-aggregated, suggesting good colloidal stability with polydispersity index (PDI) of 0.007. The very low PDI value (0.007) indicates exceptional mono-dispersity and highly controlled synthesis conditions, as PDI values below 0.1 are generally considered highly mono-disperse^26^. The low PDI values were correlated with a narrow and uniform distribution of the particle sizes^59^. The crystalline nature of the AgNPs was definitively confirmed by analyzing the selected area electron diffraction (SAED) pattern (Fig. 4f), which exhibited a well-defined diffraction lattice in the silver region, thus confirming the crystalline structure of the synthesized particles^60^.

#### Optimization of AgNPs biosynthesis

The biosynthesis of AgNPs was monitored using spectrophotometric techniques to optimize reaction conditions, including AgNO_3_ concentration, fungal biomass, pH, and the reaction temperature.

**Fig. 5:**
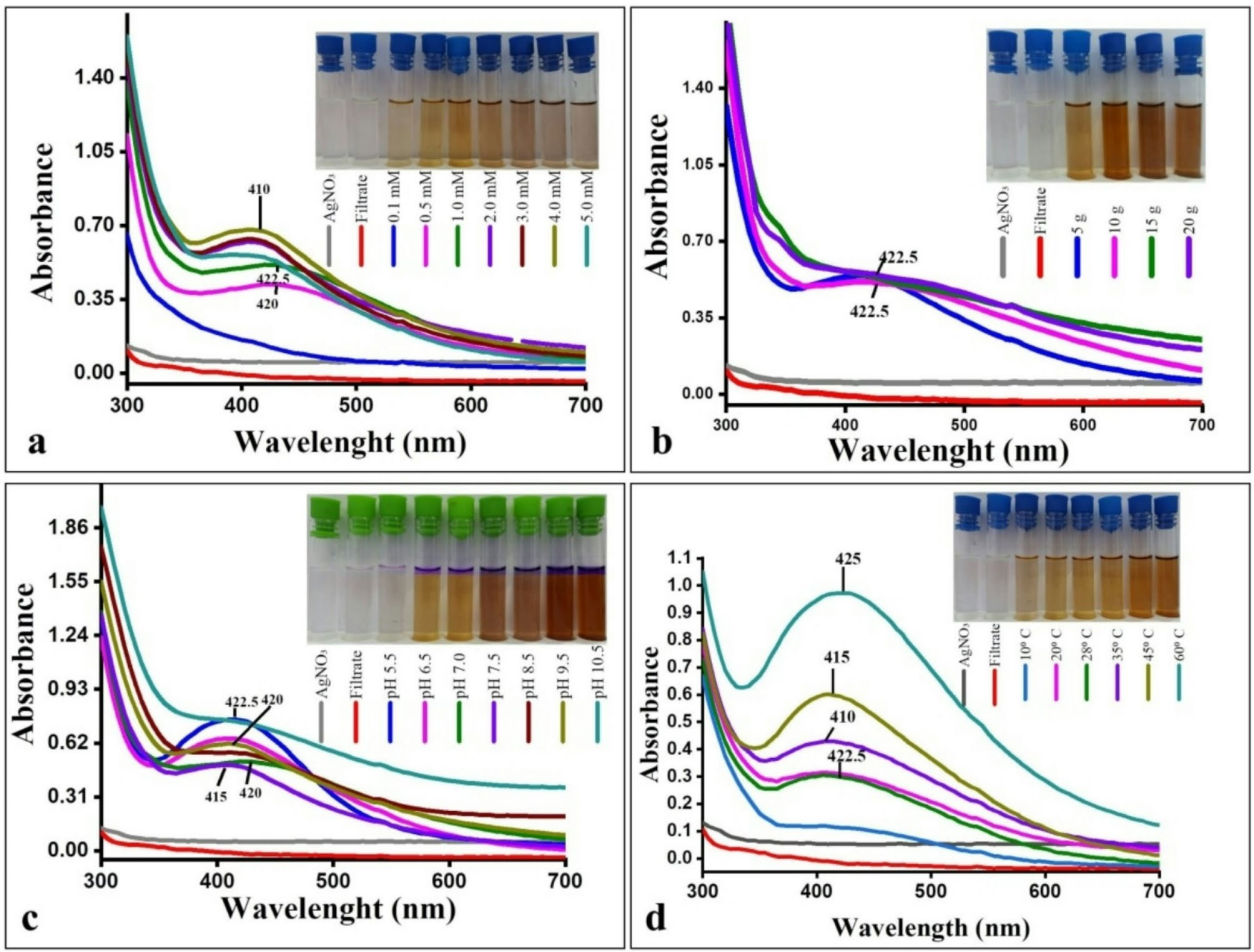
UV-visible spectra of the biosynthesized AgNPs by *T. funiculosus* at: (**a**) different AgNO_3_ concentrations, (**b**) different biomasses weights, (**c**) different pH values, (**d**) different reaction temperatures.

**Fig. 6:**
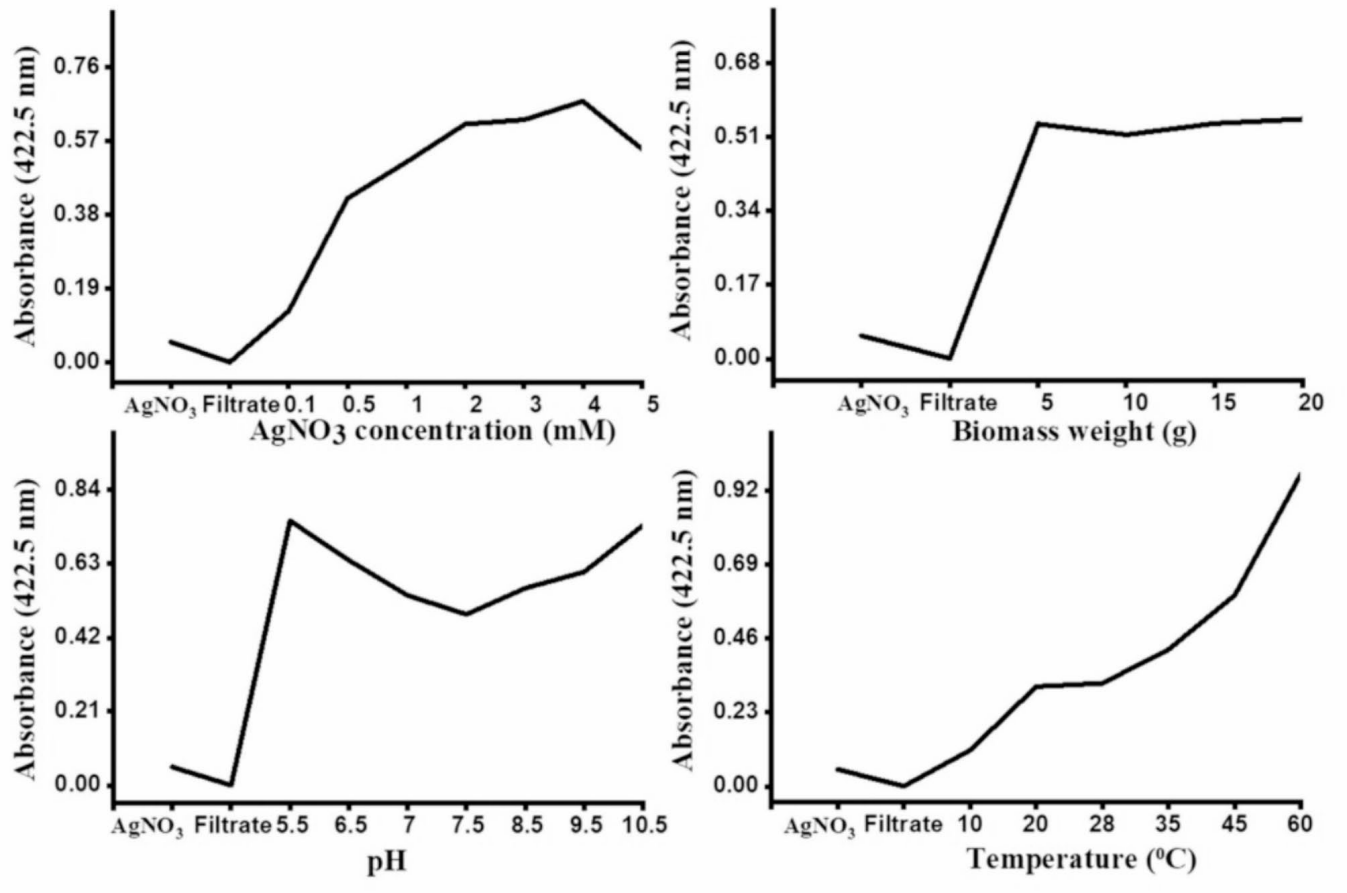
Effect of various reaction parameters on the biosynthesis of AgNPs by *T. funiculosus* with absorption observed at a wavelength of 422.5 nm.

UV-visible spectra of synthesized AgNPs at different AgNO_3_ concentration are shown in Fig. 5a. The highest intensity of the SPR band was observed at 410 nm for the 4.0 mM AgNO_3_ concentration, indicating a greater production of AgNPs. The 1 mM concentration displayed a symmetrical SPR band for the AgNPs at 422.5 nm. With increasing the AgNO_3_ concentration, the peak symmetry was distorted and flattened, indicating non-uniformity in particle size^61^. There was a gradual increase in the production of AgNPs up to 4 mM AgNO_3_ concentration, followed by a decline (Fig. 6). Utilizing AgNO_3_ concentrations higher than 4.0 mM resulted in AgNPs precipitation, leading to the absence of a typical SPR band. This decline suggested that higher AgNO_3_ concentrations led to insufficient fungal proteins for the synthesis and stabilization of AgNPs^62^. While 2, 3, and 4 mM concentrations of AgNO_3_ supported the biosynthesis of AgNPs, 1 mM was chosen for its lower toxicity and uniformity of particle size.

UV–visible absorption spectra were recorded in Fig. 5b indicating that with the biomass weight increased from 5 to 20 g, the SPR band became broader and flattened with AgNPs precipitation. The optimum wet weight of *T. funiculosus* biomass was determined to be 5 g (Fig. 6). Amount of biomass used in AgNPs biosynsesis depending on the fungus species employed. Some studies reported higher production at lower biomass concentrations^13,63^, others found higher synthesis rates at higher concentrations^64,65^. For the successful synthesis of nanoparticles it is necessary to use a balanced amount of organic materials and the amount of metal precursors^66^.

Figure 5c shows the UV-visible spectra of AgNPs synthesized at different pH. The SPR band intensity increased at pH 5.5 showing higher symmetry, confirming the uniform distribution of AgNPs. The SPR band was flattened at pH 10.5 because the protein structure was affected and lost its activity resulting in AgNPs aggregation. The results indicated high biosynthesis rates for AgNPs at pH 5.5 (Fig. 6). The optimal pH for AgNPs biosynthesis varies, with some studies suggesting an acidic mixture^10,13,67^ while others prefer an alkaline mixture^62,68,69^. This variation is due to different metabolites produced by the fungal strains^70^.

The synthesis of AgNPs was significantly influenced by increasing the temperature of the reaction mixture. The UV–visible absorption spectra indicated that with increasing temperature, the SPR band also increased (Figs. 5d and 6). It was observed that elevated temperatures accelerated the reduction process of silver ions to AgNPs^61,65^. The SPR band intensity was obtained at 60 °C indicating the highest production rate of AgNPs at this temperature.

The optimal conditions for AgNPs biosynthesis by *T. funiculosus* are 1 mM AgNO_3_, 5 g of biomass, pH 5.5, and a reaction temperature of 60 °C.

### Antimicrobial and anticancer activities of biosynthesized AgNPs and their safety evaluation

#### Antimicrobial activity

**Fig. 7:**
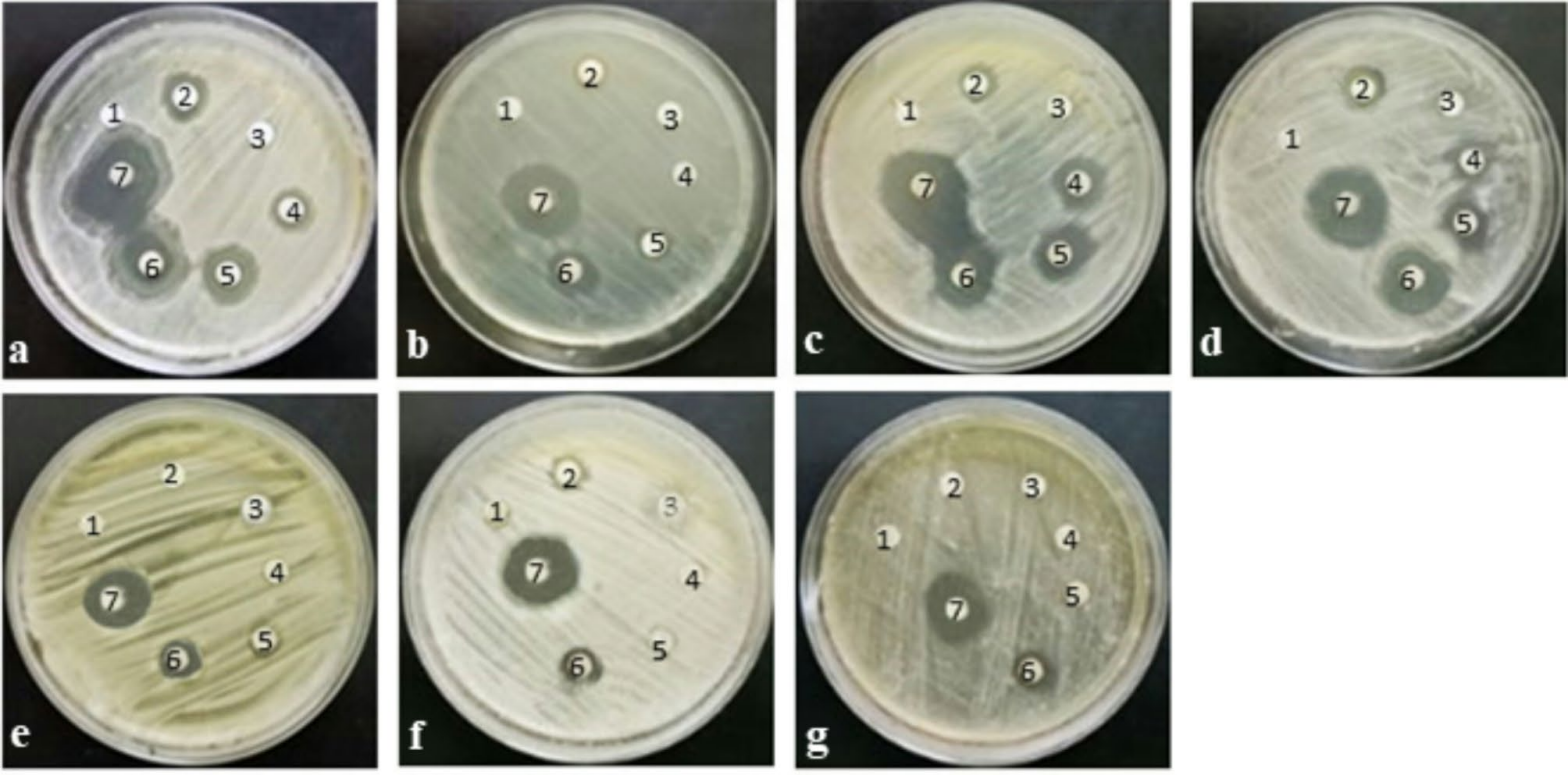
Antimicrobial activities of AgNPs: (**a**) *E. coli*, (**b**) *P. aeruginosa*, (**c**) *B. cereus*, (**d**) *S. aureus*, (**e**) *C. albicans*, (**f**) *C. tropicalis*, (**g**) *G. candidum*, (**1**) Fungal filtrate, (**2**) Positive control, (**3**) AgNO₃ (10 µg/mL), (**4**) AgNPs (5 µg/mL), (**5**) AgNPs (10 µg/mL), (**6**) AgNPs (25 µg/mL), (**7**) AgNPs (50 µg/mL).

The antimicrobial efficacy of AgNPs was evaluated against pathogenic bacteria and yeast strains (Fig. 7; Table 1). The highest antibacterial activity was observed at a concentration of 50 µg/mL, with *E. coli* (Gram-negative bacteria) exhibiting an inhibition zone diameter of 26.3 ± 0.6 mm. In contrast, *P. aeruginosa* displayed the least inhibition at the same concentration, with an inhibition zone diameter of 16.3 ± 0.3 mm. Among the tested yeast strains, *C. tropicalis* showed the highest sensitivity to AgNPs (50 µg/mL), with an inhibition zone diameter of 22.3 ± 0.3 mm. The concentration of 25 µg/mL AgNPs, within the safe daily intake range for silver, exhibited higher antimicrobial activity against all tested microorganisms compared to the control antimicrobial agents. This suggests that 25 µg/mL is a safe and effective concentration, making it a promising and applicable option for future therapeutic use. Overall, the findings demonstrated a concentration-dependent increase in the zone of inhibition, underscoring the enhanced antimicrobial efficacy of AgNPs. Several previous studies have explored the antimicrobial activity of AgNPs^71–74^. The biosynthesized AgNPs from the cell filtrate of *Fusarium oxysporum* at concentration 50 µL exhibited highly inhibitory zone against *S. aureus* (18 ± 0.66 mm) while against *C. albicans* (10 ± 0 mm) showed a moderate inhibition zone^75^. The zone of inhibition of AgNPs (40 µg/mL) biosynthesized by *Penicillium italicum* against *S. aureus*,* E. coli*,* C. albicans*, and *C. tropicalis* were 15 ± 1.3, 17 ± 0.3, 25 ± 0.5, and 24 ± 2.0 mm, respectively^76^. Ammar et al.^77^ reported that the biosynthesized AgNPs (200 µL) by *Pichia kudriavzevii* exhibited strong antimicrobial activity against *E. coli*,* S. aureus*, and *C. tropicalis* and the inhibition zone diameter were 19 ± 1.02, 20 ± 0.07, 25 ± 0.17 mm, respectively. According to Algotiml et al.^78^ the capped AgNPs (200 µL) with the *Ulva rigida* extract exhibited highly antimicrobial activity against *B. cereus*,* E. coli*,* S. aureus*, and *C. albicans* with inhibition zone diameter of 14 ± 1.0, 19 ± 1.0, 13 ± 1.0, and 13 ± 0.0 mm, respectively. Gond et al.^51^ reported that *E. coli* and *P. aeruginosa* showed inhibition zone of 14 mm when treated with 0.5 mM AgNPs biosynthesized by *Phomopsis helianthi*.

The MIC of AgNPs was determined for the tested microorganisms as detailed in Table 1. *Escherichia coli* (of bacterial strains) exhibited the highest susceptibility to AgNPs, with an MIC value of 3.7 ± 0.3 µg/mL. *Candida tropicalis* (of yeast strains) demonstrated the greatest susceptibility, with an MIC value of 6.3 ± 0.3 µg/mL. In contrast, *P. aeruginosa* and *G. candidum* exhibited the least susceptibility, with MIC values of 5.7 ± 0.3 and 13.3 ± 0.3 µg/mL, respectively. These findings are consistent with previous studies, which reported that green-synthesized AgNPs exhibited superior antibacterial activity compared to chemically synthesized AgNPs against *Staphylococcus aureus*, with MIC values of 4 and 8 µg/mL, respectively^79^. The MIC values for *E. coli*, *P. aeruginosa*, *S. aureus*, and *C. albicans* have been reported to range from 1.56 to 12.5 µg/ mL^14,63,80,81^. In addition, Saxena et al.^20^ reported that the MIC of AgNPs for *E. coli* and *S. aureus* was 100 ppm. Ribeiro et al.^49^ observed that AgNPs exhibited antifungal activity against clinical strains of *C. albicans*, *C. krusei*, *C. glabrata*, *C. parapsilosis*, *C. tropicalis*, and *C. guilliermondii*, common in hospital infections, with MIC values ranging from 1.25 to 40 µM.

**Fig. 8:**
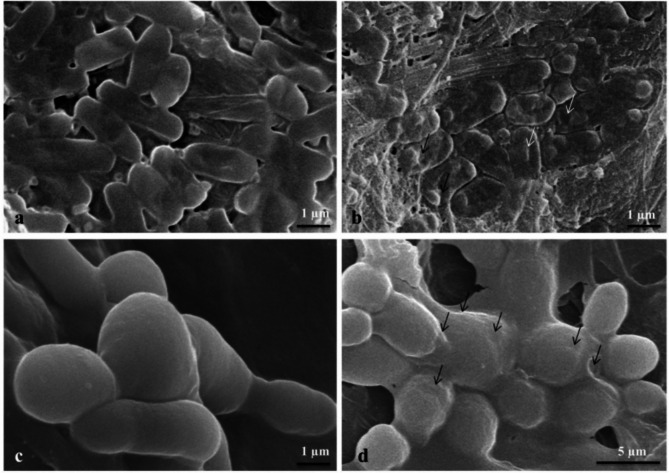
The scanning electron microscopy (SEM) images: (**a**) *E. coli* before treatment, (**b**) *E. coli* after treatment with 3.7 µg/mL AgNPs, (**c**) *C. albicans* before treatment, (**d**) *E. coli* after treatment with 7.3 µg/mL AgNPs. The arrows indicate the morphological changes in the cells.

The scanning electron microscopy (SEM) images, as shown in Fig. 8, provide detailed visual evidence of structural changes in the cells following exposure to AgNPs. These alterations include membrane disruption, surface damage, and significant changes in cell morphology, which collectively suggest the antimicrobial activity of AgNPs.

**Table 1:** Inhibitory zone diameter and MIC of the biosynthesized AgNPs (*p* < 0.05).

Microorganisms		Inhibition zone diameter in mm								MIC (µg/mL)
		Positive control		Fungal filtrate	AgNO₃ (10 µg/mL)	AgNPs (µg/mL)				
						5	10	25	50	
Gram-negative bacteria	*E. coli*	11.7 ± 0.3	Ampicillin (10 µg)/mL)	–	–	12.3 ± 0.3	14.7 ± 0.3	21.0 ± 1.0	26.3 ± 0.6	3.7 ± 0.3
	*P. aeruginosa*	7.3 ± 0.3		–	–	–	7.7 ± 0.3	12.7 ± 0.3	16.3 ± 0.3	5.7 ± 0.3
Gram-positive bacteria	*B. cereus*	8.7 ± 0.3		–	–	12.3 ± 0.3	15.0 ± 1.0	17.3 ± 0.3	23.0 ± 1.0	4.3 ± 0.3
	*S. aureus*	12.3 ± 0.3		–	–	9.0 ± 1.0	12.7 ± 0.3	17.3 ± 0.3	22.0 ± 1.0	4.7 ± 0.3
Yeast species	*C. albicans*	-	Fluconazole (25 µg/mL)	–	–	–	8.0 ± 1.0	12.3 ± 0.3	16.0 ± 1.0	7.3 ± 0.3
	*C. tropicalis*	8.7 ± 1.0		–	–	–	8.0 ± 0	14.3 ± 0.3	22.3 ± 0.3	6.3 ± 0.3
	*G. candidum*	-		–	–	–	–	11.7 ± 0.3	14.7 ± 0.3	13.3 ± 0.3

AgNPs were effective in inhibiting a wide range of microorganisms, particularly showing greater efficacy against bacterial species compared to yeast^82^. It was reported that AgNPs were more effective against Gram-negative bacteria than Gram-positive bacteria^6,83,84^. In this study, AgNPs demonstrated activity against a broad range of microorganisms. The highest and least antibacterial activities of AgNPs were observed within Gram-negative bacterial strains. The susceptibility of microorganisms to AgNPs is influenced by both the type of the microorganism and nanoparticle concentration, with antimicrobial activity also being affected by factors such as shape, size, and surface chemistry^85^. The differences in AgNPs effects are attributed to variations in cell wall composition and complexity, with simpler bacterial cells being more susceptible compared to fungal cells with robust walls and detoxification mechanisms^74^. However, the obtained results confirm the high efficacy of AgNPs in affecting these organisms compared to the antimicrobial agents such as ampicillin and fluconazole. Despite the negatively charge of both AgNPs (negative Zeta potential) and microbial cell membranes, the antimicrobial activity of AgNPs is primarily attributed to their ability to interact with the cell membrane through electrostatic and surface interactions^86^. This interaction enables AgNPs to accumulate on the bacterial surface, potentially inducing a Trojan horse effect, whereby high concentrations of antibacterial silver ions are locally released, thereby amplifying their antimicrobial efficacy^87^. The small size and high surface area of AgNPs enable them to easily penetrate cell walls and membranes, disrupting cellular processes by interacting with DNA causing structural changes that prevent replication, and binding to sulfhydryl groups on enzymes, inactivating them and inhibiting cell division^88,89^. AgNPs also induce protein denaturation, disrupt membrane integrity, and increase permeability leading to cell lysis^90^. They penetrate the outer membrane of bacterial cells, destabilize the membrane, and dissipate the proton motive force, causing further damage^69^. The small size AgNPs induce higher reactive oxygen species (ROS) production, causing oxidative stress that disrupts the mitochondrial chain and leads to superoxide anion leakage^78^. The multiple mechanisms of action of AgNPs including membrane disruption, DNA damage, enzyme inactivation, and ROS production, make them promising candidates for antimicrobial therapies^91^. However, further studies are needed to evaluate AgNPs for clinical uses.

#### Anticancer and cytotoxicity activities

**Fig. 9:**
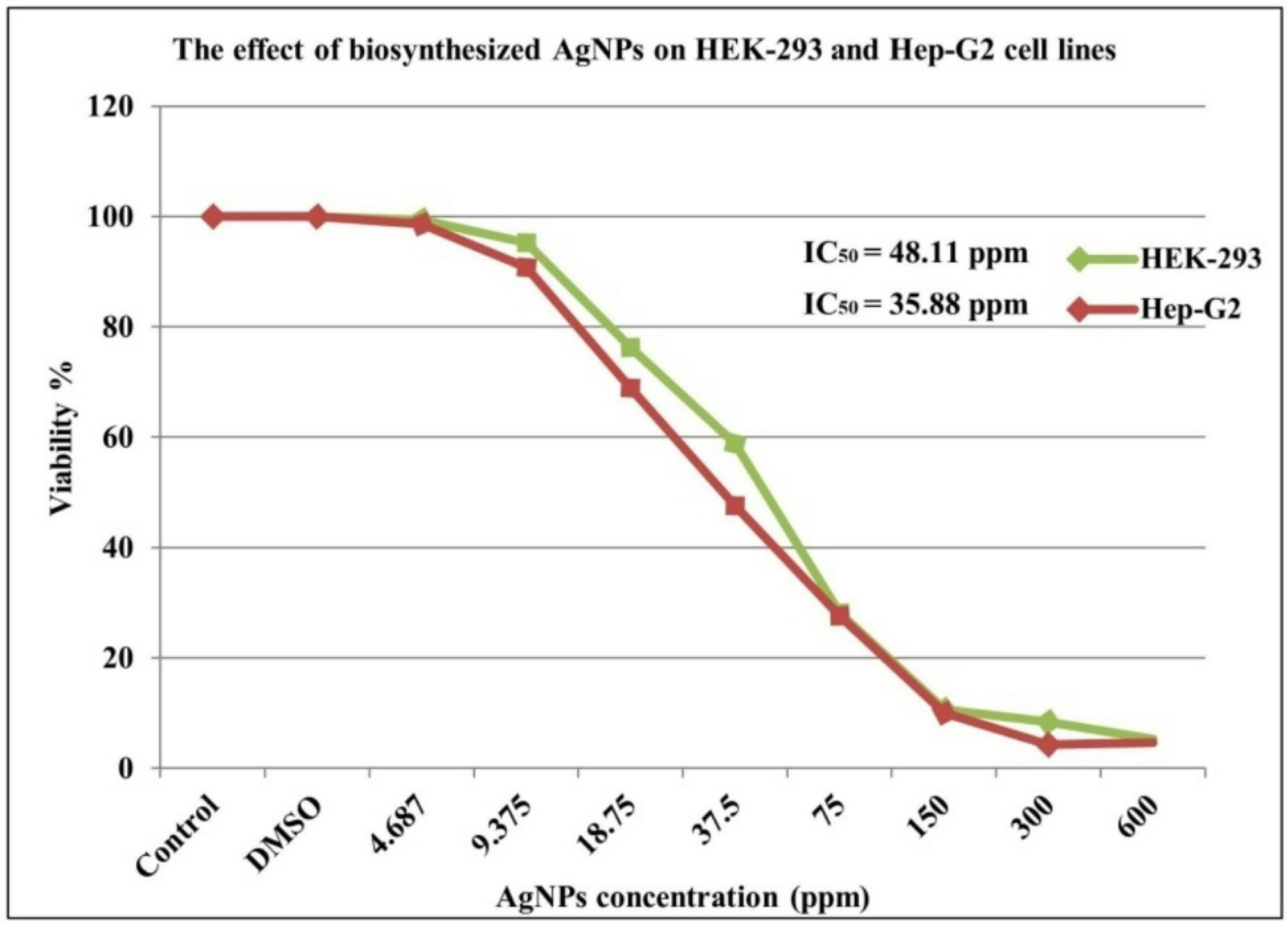
Anticancer and cytotoxic activity of AgNPs against HEK-293 and Hep-G2 cell lines, as determined by MTT assay.

**Fig. 10:**
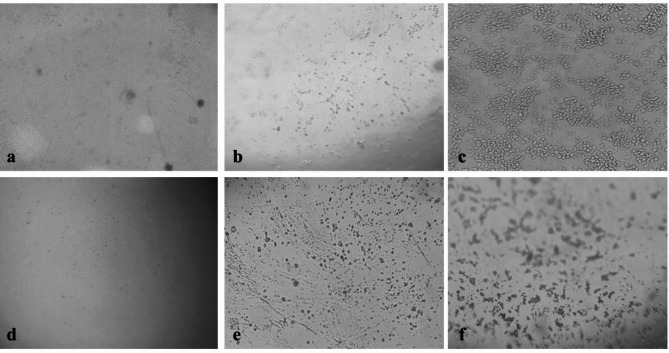
Morphological changes in the treated cell lines: (**a**) HEK-293 untreated cells, (**b**) HEK-293 cells treated with 48.11 µg/mL AgNPs, (**c**) HEK-293 cells treated with 600 µg/mL AgNPs, (**d**) Hep-G2 untreated cells, (**e**) Hep-G2 cells treated with 35.88 µg/mL AgNPs, (**f**) Hep-G2 cells treated with 600 µg/mL AgNPs.

The cell viability and cytotoxicity of the biosynthesized AgNPs were investigated *in vitr*o against human embryonic kidney cells (HEK-293, normal cell line) and liver hepatocellular adenocarcinoma (Hep-G2, cancer cell line) using MTT assay (Fig. 9). The results indicated that cytotoxicity increased with increasing AgNPs concentration. The IC_50_ values for HEK-293 and Hep-G2 cell lines were found to be 48.11 and 35.88 ppm, respectively. These findings suggest that AgNPs selectively target cancer cells while sparing normal cells, highlighting their potential as a therapeutic agent in cancer treatment. The untreated cells and those treated with DMSO showed identical results. Exposure to AgNPs induces significant morphological changes in both HEK-293 and Hep-G2 cells, including cell rounding, cytoskeletal disruption, and altered cell adhesion. However, Hep-G2 cancer cells exhibit more pronounced alterations due to their altered cellular environment, potentially reflecting a higher susceptibility to nanoparticle-induced toxicity compared to HEK2-93 cells (Fig. 10). Several studies have explored the anticancer effects of AgNPs on different cancer cell lines^4,22,92^. AgNPs demonstrated a safer profile for the treated cancer cells compared to cisplatin (cisplatin was the first FDA-approved platinum compound for chemotherapy), as evidenced by the IC_50_ values. The IC_50_ for cisplatin was 2.5145 ppm for HEK-293 cells^93^ and 2.319 ppm for Hep-G2 cells^94^. The results are consistent with those of Bhatnagar et al.^95^ who demonstrated that biosynthesized AgNPs exhibited significant efficacy against the Hep-G2 cell line with an IC_50_ of 11.1 µg/mL, while over 60% of HEK-293 cells survived exposure to 100 µg/mL of AgNPs. The results of AlBadwy et al.^96^ indicated a potent cytotoxicity of green synthesized AgNPs against Hep-G2 cell line compared to the WISH (normal cell line) with IC_50_ of 24.5 and 43 µg/mL, respectively. Khuda et al.^97^ reported an IC_50_ value of 5.38 µg/mL for AgNPs against Hep-G2 cells. Youssif et al.^98^ reported that AgNPs exhibited cytotoxicity against Hep-G2 cell line with an IC_50_ of 10.7 µg/mL, and the increased cytotoxicity in cancer cells is associated with impaired DNA repair mechanisms, which enhance their susceptibility to ROS-mediated genotoxicity. In contrast, Shahzad et al.^13^ did not observe any toxic effect of AgNPs biosynthesized by *Aspergillus fumigatus* on the Hep-G2 cell lines, which could be attributed to differences in the biological source, nanoparticle shape, capping agents, and AgNPs concentration.

AgNPs have demonstrated significant potential as anticancer agents through various mechanisms. A key factor in their cytotoxicity is their ability to induce ROS production, which induce oxidative damage to cellular components, including DNA, proteins, and lipids, ultimately leading to cell death^26^. AgNPs release silver ions that bind to thiol groups on cellular enzymes, inactivating them and disrupting essential metabolic processes^92^. They also induce DNA denaturation by breaking hydrogen bonds between nitrogenous bases, impairing cell replication and division^55^. Furthermore, AgNPs interfere with cellular respiration and ATP production, leading to mitochondrial dysfunction and triggering apoptosis^99^. AgNPs also inhibit angiogenesis, which is crucial for tumor growth and metastasis, allowing them to target cancer cells^95^. The presence of bioactive molecules on the surface of AgNPs, along with factors such as particle size, shape, surface modifications, and cancer type, can synergistically enhance their anticancer activity^100^. AgNPs exhibit minimal toxicity to humans at low concentrations, their selective action on cancer cells makes them promising candidates for future cancer therapies.

#### Antioxidants activities of AgNPs

**Fig. 11:**
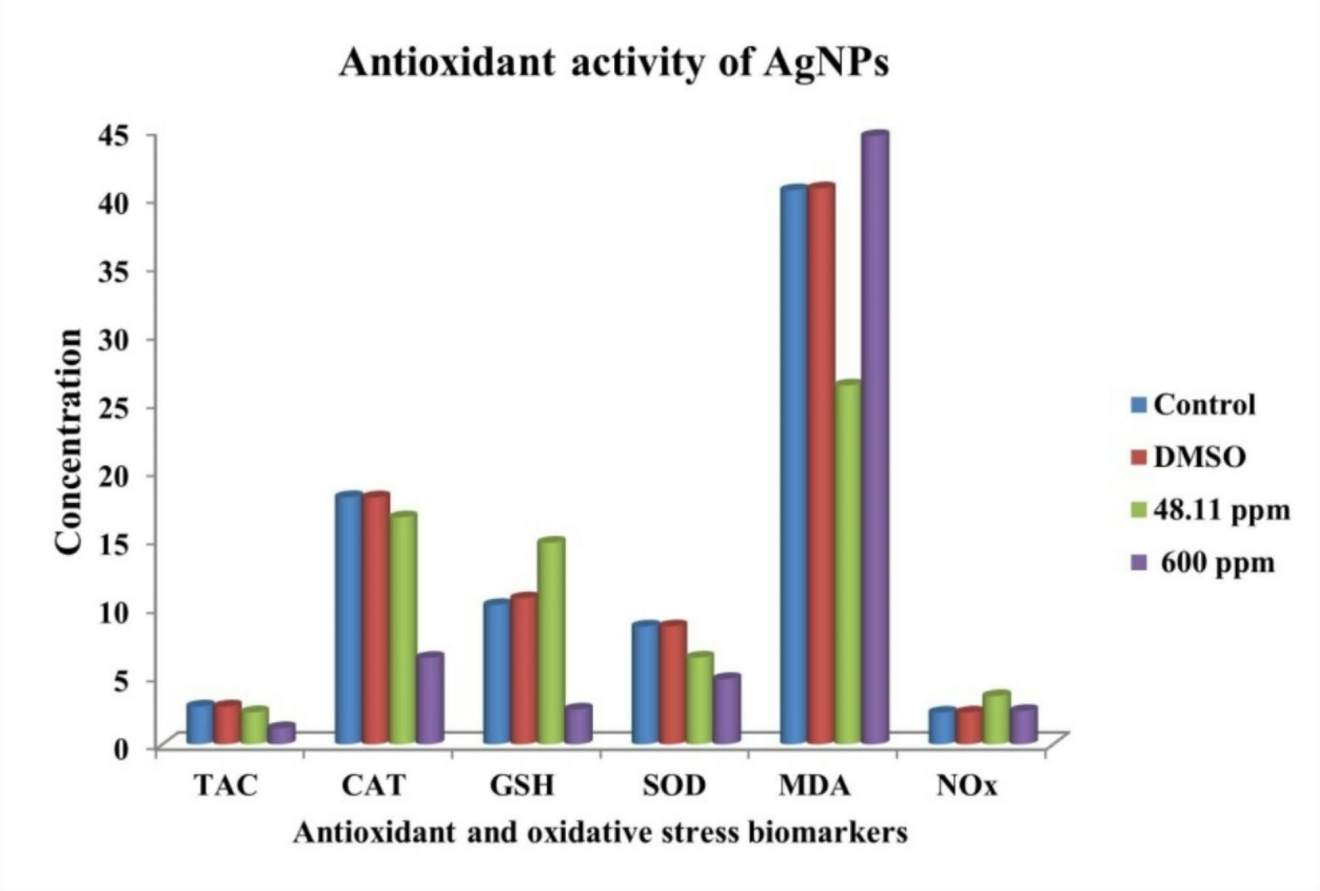
Antioxidant properties and oxidative stress induction by AgNPs.

Reactive oxygen species are generated as a byproduct of normal aerobic metabolism. These unstable free radicals can harm cellular components such as lipids, proteins, and DNA, leading to various diseases. Organisms have evolved intricate antioxidant systems to counteract ROS and minimize their damage^101^. This study assessed the impact of AgNPs on crucial antioxidant and oxidative stress biomarkers. The data presented in Fig. 11; Table 2 indicates that at AgNPs of 48.11 ppm (IC_50_) led to a slight decrease in TAC (from 2.77 to 2.35 mM/L), CAT (18.1 to 16.62 U/g tissue), and SOD (8.63 to 6.35 U/g tissue) levels, along with an increase in GSH (from 10.29 to 14.76 mmol/g) levels when compared with the untreated cell. Conversely, at 600 ppm, AgNPs reduced antioxidant activity across the measured parameters. The effect of AgNPs on oxidative stress parameters demonstrated a variable response. Specifically, MDA levels significantly decreased at 48.11 ppm AgNPs, from 40.57 nmol/mL in the control cells to 26.28 nmol/mL. However, at 600 ppm, MDA levels increased to 44.54 nmol/mL. Nitric oxide levels were higher at 48.11 ppm (3.53 µmol/L) than 600 ppm (2.44 µmol/L). The study conducted by Waly et al.^102^ investigated the effects of cisplatin on HEK-293 cells, focusing on antioxidant and oxidative stress biomarkers. The study revealed that cisplatin at a concentration of 0.3 ppm resulted in a TAC of 11.87 mM/L, CAT activity of 58.19 U/g, and SOD activity of 15.22 U/mg. Also, cisplatin treatment led to a significant depletion of intracellular GSH levels and a marked increase in MDA levels. A study by Shinde et al.^103^ reported that cisplatin induced significant oxidative stress, as evidenced by a marked increase in MDA levels and a reduction in GSH levels. This indicated that AgNPs showed a safer therapeutic profile, but efforts are needed to improve their safety in in vivo testing. Numerous studies have investigated the influence of AgNPs on antioxidant and oxidative stress biomarkers^76,97^. Similarity, El-Rafie & Hamed^104^, reported that AgNPs have a significant antioxidant effect by increasing GSH level and reducing MDA levels. In a study by El-Sonbaty^105^ AgNPs showed a slightly decrease in SOD (from 4.2 to 4.1 µg/g protein) and CAT (from 3.5 to 3.1 µg/g protein) activities, as well as slightly increase GSH levels (from 52.9 to 54.8 µg/g protein), along with increased NO (from 4.1 to 5.3 µM/g protein) and MDA (from 9.0 to 10.2 µM/g protein) levels. Ranjbar et al.^106^ investigated the effects of AgNPs at different concentrations (5, 50, 250, and 500 mg/kg) in rat plasma, with the 500 mg/kg dose enhancing the activities of CAT and SOD while decreasing total antioxidant capacity TAC, indicating that the antioxidant properties of AgNPs are dose-dependent. In contrast, Erjaee et al.^107^ reported that the biosynthesized AgNPs by *Chamaemelum nobile* led to a decrease in CAT (from 13.54 to 8.39 U/mg protein), and SOD (from 3.64 to 3.31 U/mg protein) activities, accompanied by an increase in MDA levels (from 159.64 to 181.6 mmol/mg protein).

The antioxidant mechanism of AgNPs is attributed to the silver ability to exist in two oxidation states (Ag^+^ and Ag^2+^), enabling AgNPs to quench free radicals by either donating or accepting electrons, depending on the reaction conditions^101^. The biological activities and environmental impact of nanoparticles are significantly influenced by capping agents, which modify their physicochemical properties through steric effects on the nanoparticle surface^108^. Nirmala & Sridevi^109^ explained that the capping agent of AgNPs with proteins could improve their ability to exhibit antioxidant properties. The antioxidant potential of AgNPs is attributed to the functional groups on their surface, which can help counteract the produced free radicals^110–112^. At higher concentrations of AgNPs, the antioxidant enzyme activity is affected by the interaction between enzyme proteins and capping proteins of AgNPs. The changes in protein conformation and AgNPs dissolution depended on protein structure, leading to varying degrees of enzymatic activity modulation^113^. The antioxidant properties of AgNPs at low concentrations make them suitable and safe for therapeutic interventions that combat oxidative damage, supporting their potential in medical and pharmaceutical applications.

**Table 2:** Antioxidants and oxidative stress activities of AgNPs (*p* < 0.05).

AgNPs	Antioxidants biomarkers				Oxidative stress biomarkers	
	TAC (mM/L)	CAT (U/g tissue)	GSH (mmol/g)	SOD (U/mg tissue)	MDA (nmol/ml)	NOx (µmol /L)
Untreated cells	2.77 ± 0.013	18.1 ± 0.0133	10.29 ± 0.032	8.63 ± 0.029	40.57 ± 0.081	2.32 ± 0.012
DMSO	2.77 ± 0.002	18.08 ± 0.035	10.69 ± 0.17	8.64 ± 0.001	40.71 ± 0.023	2.33 ± 0.006
IC_50_ (48.11 ppm)	2.35 ± 0.006	16.62 ± 0.006	14.76 ± 0.01	6.35 ± 0.006	26.28 ± 0.077	3.53 ± 0.01
600 ppm	1.18 ± 0.019	6.33 ± 0.028	2.55 ± 0.028	4.78 ± 0.009	44.54 ± 0.045	2.44 ± 0.013

### Inflammatory and anti-inflammatory activities of AgNPs

**Fig. 12:**
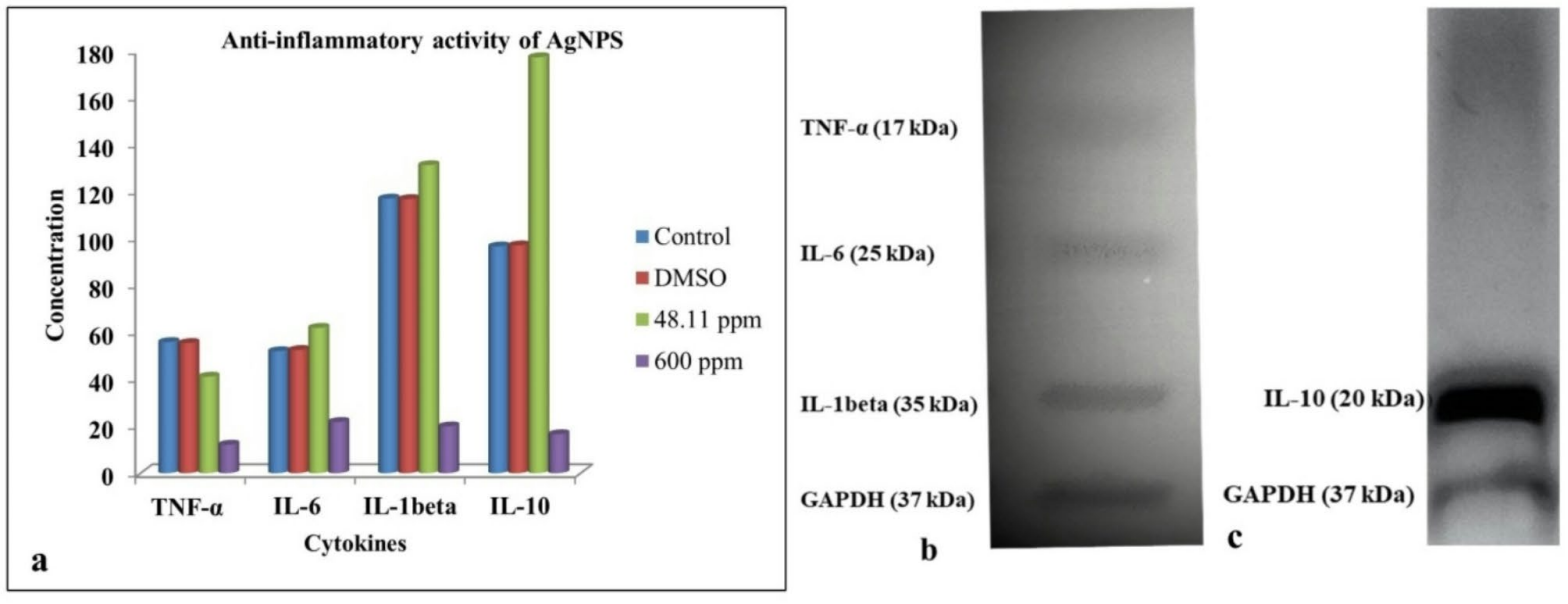
Effect of AgNPs on cytokines expression in HEK-293 cell line: (**a**) Quantification of cytokine expression by ELISA, (**b**) Western blot image showing the protein expression of inflammatory cytokines (TNF-α, IL-6, and IL-1β), (**c**) Western blot image showing the protein expression of anti-inflammatory cytokine (IL-10).

Cytokine production is a crucial event in the regulation of inflammatory response, and recent research has increasingly focused on the potential of biosynthesized nanoparticles as selective inhibitors of cytokine activity^114^. In this study, the HEK-293 cell line treated with AgNPs displayed varying inflammatory and anti-inflammatory responses, as detailed in Fig. 12a; Table 3. The treated cells with the IC_50_ (48.11 ppm) concentration showed a decrease in TNF-α level (from 55.77 to 41.06 ng/L), while IL-1β (from 116.84 to 131.07 pg/mL) and IL-6 (from 51.89 to 61.84 ng/L) exhibited an increase. Conversely, all tested inflammatory cytokines demonstrated a significant decline when the cell line was treated with 600 ppm AgNPs. The anti-inflammatory cytokine IL-10 significantly increased (from 96.47 to 177.0 pg/mL) in the cells treated with the 48.11 ppm but decreased substantially at 600 ppm (from 96.47 to 16.56 pg/mL), suggesting the anti-inflammatory properties of AgNPs at low concentrations. The obtained results were corroborated by Western blot analysis, which revealed that HEK-293 cells treated with 48.11 ppm of AgNPs exhibited a reduction in inflammatory cytokines expression and an increase in anti-inflammatory cytokine levels as shown in Fig. 12b, c (Western blot analysis data and full gel images are provided in the supplementary information file). The band density of the cytokines was significantly reduced compared to that of the GAPDH control protein. In comparison, Shinde et al.^103^ reported that cisplatin (6 ppm) treatment significantly increased TNF-α levels to 49.86 pg/mg protein in Hek-293 cells, and in vivo administration in rats led to significant increases in TNF-α (75.55 pg/mg protein), IL-6 (87.70 pg/mg protein), and IL-1β (107.0 pg/mg protein) in kidney tissue homogenates, compared to normal rats (TNF-α 30.32, IL-6 22.82, IL-1β 37.67 pg/mg protein). These findings highlight the contrasting inflammatory effects of AgNPs and cisplatin, further supporting the potential anti-inflammatory properties of AgNPs. In a study by Bold et al.^115^ using a burn injury murine model, the treatment group with AgNPs exhibited significantly lower expression levels of TNF-α, IL-1β, and IL-6 compared to the control group alongside elevated IL-10 expression. IL-10, an anti-inflammatory cytokine, helps regulate inflammation during wound healing by inhibiting the production of pro-inflammatory cytokines, including TNF-α, IL-1β, and IL-6. Alwan & Al-Saeed^116^ found that AgNPs have an anti-inflammatory effect in polycystic ovarian syndrome rats by reducing the concentrations of inflammatory cytokines TNF-α, IL-6, and IL-18. Tyavambiza et al.^117^ reported that AgNPs demonstrated anti-inflammatory activity by inhibiting the secretion of pro-inflammatory cytokines (TNF-alpha, IL-6, and IL-1 beta) in lipopolysaccharide-treated macrophages. The inhibition of TNF-α expression by AgNPs may offer a cost-effective approach for anticancer therapy and the treatment of other inflammation-related illnesses^118^.

**Table 3:** Inflammatory and anti-inflammatory activities of AgNPs, with statistical significance (*p <* 0.05), as determined by ELISA.

AgNPs	Inflammatory cytokines			Anti-inflammatory Cytokine
	TNF-α (ng/L)	IL-6 (ng/L)	IL-1beta (pg/ml)	IL-10 (pg/ml)
Untreated cells	55.77 ± 0.33	51.89 ± 0.36	116.84 ± 0.199	96.47 ± 0.24
DMSO	55.36 ± 0.74	52.4 ± 0.43	116.63 ± 0.72	96.93 ± 0.66
IC_50_ (48.11 ppm)	41.06 ± 0.15	61.84 ± 0.13	131.07 ± 0.13	177.0 ± 0.24
600 ppm	12.10 ± 0.14	21.79 ± 0.39	19.89 ± 0.06	16.56 ± 0.32

The topical treatment with AgNPs in skin wound healing models significantly reduced IL-6 levels and stimulated the production of IL-10^119^. AgNPs control inflammation through multiple mechanisms, including modulating cytokine release, inhibiting inflammatory mediators, regulating oxidative stress, suppressing NF-κB signaling, and influencing immune cells^120^. AgNPs selectively inhibit COX-2 by inducing structural changes that block prostaglandin production and suppress NF-κB signaling, reducing leukocyte chemotaxis and inflammation^121,122^. AgNPs can influence the immune system by regulating the production of pro-inflammatory cytokines, such as TNF-α, IL-6, and IL-1β^107^. They interact with immune cells like macrophages, shifting their response from pro-inflammatory to anti-inflammatory, potentially mitigating tissue damage and disease progression in chronic inflammation^123^. This modulation helps restore immune balance, reduce oxidative stress, and minimize tissue damage^124^. Consequently, AgNPs may present promising therapeutic strategies for inflammatory disease management, with their effects being dose-dependent.

Finally, the obtained results in anticancer, antioxidant, and anti-inflammatory studies promoted the safety and therapeutic potential of AgNPs at low doses. AgNPs exhibited significant cytotoxicity against cancer cells while sparing normal cells, highlighting their selective action. Their antioxidant activity was demonstrated by modulation of oxidative stress biomarkers, supporting their role in reducing cellular damage. Furthermore, AgNPs induced the production of the anti-inflammatory cytokine IL-10, while suppressing pro-inflammatory cytokine (TNF-α), indicating their potential to modulate inflammation effectively. These findings suggest that AgNPs could serve as safe and effective therapeutic agents for cancer, oxidative stress, and inflammation. However, further in vivo studies are necessary to assess their long-term safety and efficacy for clinical applications.

## Materials and methods

### Sample collection, isolation and morphological study

The endophytic fungus *Talaromyces funiculosus* was isolated from the leaves of the medicinal plant *Euphorbia hirta* L., which were collected from Wadi Bir-EL-Ain in the eastern desert of Sohag Governorate, Egypt. The isolation of the fungal strain followed the method outlined by Hallmann et al.^125^. Further purification was carried out through sub-culturing on potato dextrose agar (PDA; Oxoid, Basingstoke, England). Morphological characteristics were observed and captured by a digital camera (Toup Tek XCAM1080PHA Toup Tek, Zhejiang, China) mounted on an Olympus BX51 (Olympus, Tokyo, Japan) compound microscope. The fungus was identified through morphological and microscopic observations following the method described by Yilmaz et al.^39^. It was grown on Czapek yeast extract agar (CYA), malt extract agar (MEA; 2% w/v), PDA, creatine sucrose agar (CREA), and incubated at 25 ˚C and 30 ˚C for 7 days. The fungal culture is deposited in Sohag University microbial culture collection, Egypt (SUMCC 22011).

### DNA extraction, sequencing and phylogenetic analyses

Cultures grown on glucose and yeast with peptone (GPY)^126^ were used for genomic DNA extraction using the Microbial DNA Extraction Kit (MOBIO; Mo Bio Laboratories, Carlsbad, CA, USA) following the manufacturer’s instructions. The primer pairs ITS1/ITS4^127^ were used for amplification of the DNA sequences of the internal transcribed spacers (ITS). PCR reactions, cycling parameters and sequencing were done following Abdel-Wahab et al.^128^ by Solgent Co. Ltd (South Korea).

Sequences were assembled using Sequencher 4.2.2 (Gene Codes Corporation) and aligned with relevant ones retrieved from GenBank using ClustalX^129^. Phylogenetic analyses were conducted using Maximum Parsimony (MP) and Maximum Likelihood (ML). Maximum likelihood (ML) analysis was performed using RAxMLGUI v. 2.0.8^130^ with 1000 rapid bootstrap replicates under the GTR + GAMMA substitution model. Phylogenetic analyses were performed based on details outlined by Abdel-Aziz & Bakhit^131^. Obtained sequence of our isolate was deposited in NCBI GenBank.

### Extracellular biosynthesis of AgNPs by *T. funiculosus*

*Talaromyces funiculosus* was cultured in 100 mL of malt, glucose, yeast extract, and peptone (MGYP) broth in a 250 mL Erlenmeyer flask^83^. After incubation, the mycelial biomass was harvested and washed with sterilized deionized water to remove residual culture medium components^132^. Approximately 10 g (wet weight) of the biomass was then transferred to a 250 mL Erlenmeyer flask containing 100 mL of sterilized deionized water. This flask was incubated for an additional 72 h at 28 ± 2°C^49^. The fungal filtrate was obtained by filtering the solution through Whatman filter paper No. 1. The filtrate was subsequently reacted with silver nitrate (AgNO₃) to achieve an overall silver ion concentration of 1 mM^133^. The biosynthesis reaction was conducted in the dark at 28 ± 2 °C. The fungus filtrate and the AgNO_3_ solution were used as controls under similar experimental conditions, and the color change was observed for up to 72 h^19^. The experiment was conducted in triplicate to ensure the reliability of the results and repeated three times at different intervals using distinct *T. funiculosus* batches to confirm the consistent ability of the fungus to biosynthesize AgNPs. The synthesized AgNPs were stored at room temperature for six months to assess their stability.

### Characterization of the biosynthesized AgNPs

The UV–visible spectra were recorded using a UV–visible spectrophotometer (JENWAY 7315, UK) in the wavelength range of 300–700 nm to ensure the presence of specific surface Plasmon resonance (SPR) peak of AgNPs. Deionized water was used as a blank to adjust the baseline^19^. Measurements were repeated after six months to evaluate the long-term stability of the AgNPs.

The X-ray diffraction (XRD) analysis of AgNPs powder was conducted in the 2θ range of 30° to 80° using X-ray diffractometer (D8 Advance, Germany) operating at 40 mA and 40 kV with Cu Kα radiation (λ = 1.54060 Å). The sample powder was prepared according to the method described by Neciosup-Puican et al.^134^.

The size distribution and average size of the AgNPs were determined through dynamic light scattering (DLS) measurements. The Zeta potential is the electrical potential recorded at the interface between particles and the surrounding fluid, which is specific to a charged surface and predicts the long-term stability of a colloidal dispersion^59^. The Zeta potential (surface charge) was measured using a Malvern Zetasizer Nano ZS instrument (Malvern, UK) to evaluate the stability of the nanoparticle suspensions (DLS, Zeta potential, Microanalytical Center, Cairo University, Egypt).

Fourier-transform infrared spectroscopy (FTIR) of the AgNPs and fungal filtrate were performed using a Platinum-ATR FTIR spectrometer (Bruker Alpha, Germany) in the range of 4000–400 cm⁻¹. This spectral range allowed for the investigation of molecular vibrations associated with the various functional groups on the nanoparticle surface^30^. This analysis aimed to identify the potential biomolecules responsible for the reduction, capping, and stabilization of the biosynthesized AgNPs.

High-resolution transmission electron microscope (HR-TEM) and selected area electron diffraction (SAED) analyses were conducted on the biosynthesized AgNPs using the JEOL JSM 100CX TEM instrument, Japan (TEM, Electron Microscope Unit, Cairo University, Egypt). TEM images of the nanoparticles were captured, and the polydispersity index (PDI) was subsequently calculated^29^.

### Optimization of AgNPs biosynthesis

AgNPs were produced under different physicochemical conditions to determine the optimum conditions for stable and large-scale production of AgNPs with the smallest size range. The optimization of AgNPs biosynthesis involved examining the following factors: AgNO_3_ concentration, biomass concentration pH value, and the reaction temperature. Each factor was tested by varying only a single parameter at a time. Different AgNO_3_ concentrations (0.1, 0.5, 1, 2, 3, 4, and 5 mM AgNO_3_), fungal biomass weights (5, 10, 15, and 20 g), pH values (5.5, 6.5, 7.0, 7.5, 8.5, 9.5, and 10.5), and the reaction temperatures (10, 20, 28, 35, 45, and 60˚C) were investigated. The absorbance of the colored solution that resulted from the reaction for each factor was measured by a UV-visible spectrophotometer.

### Antimicrobial and anticancer activities of the biosynthesized AgNPs and study their safety

#### Antimicrobial activity

The antimicrobial activities of AgNPs were evaluated using the Kirby-Bauer disk diffusion method as described by Hudzicki^135^. The synthesized AgNPs were tested against human pathogens: Gram-negative bacteria; *Escherichia coli* and *Pseudomonas aeruginosa*, Gram-positive bacteria; *Bacillus cereus* and *Staphylococcus aureus* (ACCB 136), and 3 pathogenic yeast; *Candida albicans* (AUMC 10440), *Candida tropicalis* (AUMC 10442), and *Galactomyces candidum* (AUMC 10443). The test was performed using Mueller-Hinton agar (MHA) for bacterial strains and Sabouraud’s dextrose agar (SDA) media for yeast strains. Four AgNPs concentrations (5, 10, 25, and 50 µg/mL) were loaded on sterile filter paper disk. All tested AgNPs concentrations were chosen based on the typical daily intake of silver from natural sources, such as food and water, which ranges from approximately 0.4 to 30 µg^22^, except for one higher concentration (50 µg/mL). This range was selected to reflect environmentally relevant concentrations, providing a basis for evaluating the potential biological effects of AgNPs within a realistic exposure range. Fungal filtrate and AgNO_3_ (10 µg/mL) disk were used as a negative controls. Ampicillin (10 µg/mL) for bacterial strains and fluconazole (25 µg/mL) for yeast strains were tested as a positive controls^10^. The formed plates were incubated at 37 °C for 24 h followed by measuring the formed inhibition zone in mm. These assays were carried out in triplicates to confirm the estimated inhibition zone.

The minimum inhibitory concentration (MIC) of AgNPs was determined for the tested pathogens by broth microdilution method using 96-well microtiter plates according to the principles described by Kowalska-Krochmal & Dudek-Wicher^136^. The concentrations of AgNPs were adjusted from 0.5 to 50 µg/mL to facilitate the MIC evaluate. Resazurin (a weakly fluorescent blue dye) was used as an indicator to determine the MIC. From the above assay, an inoculum was taken from each well that showed no visual growth and spotted on MHA/SDA plates to validate the MIC assay. All the experiments were performed in triplicate.

The effect of AgNPs on *E. coli* and *C. albicans* before and after 6 h of exposure to the MICs (3.7 µg/mL and 7.3 µg/mL, respectively), were examined using scanning electron microscopy (SEM). The tests were conducted following the method described by Rizwana et al.^74^. Microphotographs were captured, and morphological alterations were thoroughly studied compared to the untreated control cells.

#### Cytotoxicity and anticancer activities of AgNPs

The viability and cytotoxicity of AgNPs were *in vitr*o tested against human embryonic kidney cells (HEK-293, normal cell line) and liver hepatocellular adenocarcinoma (Hep-G2, cancer cell line). The tested cell lines were obtained from the American Type Culture Collection (ATCC, Microbiology Department, Faculty of Medicine, Al-Azhar University, Cairo, Egypt). The viability and cytotoxicity of AgNPs were assessed after 24 h using MTT (3-(4,5-dimethyl thiazol-2-yl)-2,5-diphenyl tetrazolium bromide) assay as described by Mosmann^137^. Briefly, the 96-well tissue culture plate was inoculated with 100 µL/well (1 × 10^5^ cells/mL) and incubated at 37 °C for 24 h to develop a complete monolayer sheet. The growth medium was decanted from 96 well microtiter plates after a confluent sheet of cells was formed. AgNPs at varying concentrations (600, 300, 150, 75, 37.5, 18.75, 9.375, and 4.687 ppm) were added to the seeded cell lines (100 µL of each concentration) and treated for 24 h at 37 °C. Cells were checked for any physical signs of toxicity (partial or complete loss of the monolayer, rounding, shrinkage, or cell granulation). MTT (20 µL) solution was added to each well. The plate was again incubated (37 °C and 5% CO2) for 1 to 5 h to allow the MTT to be metabolized. The media was dumped off and the formazan (MTT metabolic product) was re-suspended in 200 µL dimethyl sulfoxide (DMSO). The optical density was read at 560 nm and subtracted at 620 nm using a microplate reader (Stat Fax 2100, USA). The optical density was directly correlated with the cell quantity. Cytotoxicity percentage was determined using the following equation:

$${\text{Viability }}\left( \% \right){\text{ }}={\text{ }}\left( {{\text{Test OD}}/{\text{Control OD}}} \right){\text{ }} \times {\text{ 1}}00$$

$${\text{Cytotoxicity }}\left( \% \right)\,=\,{\text{1}}00{\text{ }}--{\text{ Viability }}\left( \% \right).$$

The IC_50_ value was determined as the concentration of the AgNPs required to reduce the absorbance to half that of the control. All assays were performed in triplicate. DMSO was used as a negative control, along with untreated cell lines, to evaluate the anticancer activity and cytotoxicity of AgNPs. Morphological alterations in HEK-293 and Hep-G2 cells following AgNPs treatment were assessed through microscopic imaging, offering a visual comparison between untreated and AgNPs-exposed cells.

#### Antioxidants activities of AgNPs

The effect of AgNPs on the antioxidants and oxidative stress biomarker activities in the treated HEK-293 cell line were investigated after 24 h of incubation. The levels of antioxidants and oxidative stress biomarkers were assessed at 48.11 and 600 ppm, representing the IC_50_ and the highest AgNPs concentration, respectively, after 24 h of treatment. DMSO was used as a negative control, along with untreated cell lines, to evaluate the effects of the AgNPs. The investigated antioxidants included total antioxidant capacity (TAC), catalase (CAT), reduced glutathione (GSH), and superoxide dismutase (SOD) activity. The tested oxidative stress biomarkers were malondialdehyde (MDA) and nitric oxide (NO). All tests were performed in triplicate using the enzymatic colorimetric method and read by a microplate reader (Stat Fax 2100, USA), following the protocols provided with the kits. These kits were provided by Biodiagnostic Company, Egypt and the catalog numbers for the TAC, CAT, GSH, SOD, MDA, and NO kits were TA 25 13, CA 25 17, GR 25 11, SD 25 21, MD 25 29, and NO 25 33, respectively.

#### Inflammatory and anti-inflammatory activities of AgNPs

The inflammatory cytokines, including human tumor necrosis factor-alpha (TNF-α), human interleukin 6 (IL-6), and human interleukin 1beta (IL-1β), as well as the anti-inflammatory cytokine human interleukin 10 (IL-10), were quantified using enzyme-linked immunosorbent assay (ELISA) kits from BT LAB Company, China. The assays were performed according to the manufacturer’s instructions. The cytokines were assessed at concentrations of 48.11 and 600 ppm, corresponding to the IC_50_ and the highest concentration of AgNPs, respectively after 24 h of treatment. DMSO was used as a negative control, along with untreated cell lines, to evaluate the effects of the AgNPs. The results were measured by a microplate reader (Stat Fax 2100, USA) and compared with those from the control cells. All tests were conducted in triplicate. The ELISA kits for the cytokines references were E0082Hu, E0090Hu, E0143Hu, and E0102Hu for TNF-α, IL-6, IL-1β, and IL-10 assays, respectively. Western blot analysis was performed to evaluate the protein expression levels of inflammatory cytokines and the anti-inflammatory cytokine IL-10 following treatment with AgNPs at the IC_50_ (48.11) concentration^138^. GAPDH (37 kDa) was used as a control protein. The analysis was conducted at the Microanalytical Center, Cairo University, Egypt.

### Statistical analysis

All experiments were carried out in triplicate and the results were presented as mean ± standard deviation^69^. The data were statistically analyzed by one-way analysis of variance (ANOVA) using XLSTAT version 2023.2.0 software^139^.

## Conclusion

Silver nanoparticles have widespread applications, particularly in biomedicine. In this study, the endophytic fungus *T. funiculosus* isolated from *E. hirta*, demonstrated efficient AgNPs biosynthesis, offering an environmentally friendly bioprocess with potential for biomedical applications. The biosynthesized AgNPs were spherical crystalline, stable (6 months), and mono-dispersed (PDI: 0.007), exhibiting SPR at 422.5 nm, average diameter of 34.32 nm, and Zeta potential of -18.41 mV. XRD and TEM analyses confirmed their crystalline structure, while FTIR identified a capping protein enhancing their stability. The production of AgNPs for large-scale synthesis was optimized, which greatly improved the yield, efficiency, and process consistency. The optimal biosynthesis conditions by *T. funiculosus* included 1.0 mM AgNO_3_, 5 g biomass, pH 5.5, and a temperature of 60 °C. AgNPs exhibited potent antimicrobial activity against pathogenic bacteria and yeast, with concentration-dependent effects. The concentration of 25 µg/mL AgNPs, within the safe daily intake range for silver, exhibited higher antimicrobial activity against all tested microorganisms compared to the control antimicrobial agents. This suggests that 25 µg/mL is a safe and effective concentration, making it a promising and applicable option for future therapeutic use. Cytotoxicity assays revealed higher activity against Hep-G2 (IC_50_ of 35.88 ppm) cancer cells than HEK-293 (IC_50_ of 48.11 pm) normal cells, indicating potential for cancer therapy. Additionally, AgNPs displayed significant antioxidant effects, increasing GSH and reducing MDA levels. They also suppressed the pro-inflammatory cytokine TNF-α while enhancing anti-inflammatory IL-10 production. These findings suggest that AgNPs biosynthesized by *T. funiculosus* are promising therapeutic agents. Future research should focus on evaluating the efficacy of these AgNPs in vivo studies to better understand their potential therapeutic applications. The employment of surface modifications with biocompatible coatings will enhance nanoparticle stability, minimize tissue accumulation, and promote their efficient clearance from the body. Furthermore, testing the synthesized AgNPs against drug-resistant microbial strains will be critical in assessing their viability as an alternative antimicrobial agent, potentially addressing the growing issue of antimicrobial resistance.

## Electronic supplementary material

Below is the link to the electronic supplementary material.

Supplementary Material 1

## Data Availability

All data generated or analyzed during this study are included in this published article. Sequence data that support the findings of this study have been deposited in NCBI GenBank with the accession number: PQ555628.
