# Supplementary material for: Biosynthesis of silver nanoparticles by Talaromyces funiculosus for therapeutic applications and safety evaluation
Source: Sci Rep. 2025 Apr 21;15:13750. doi: 10.1038/s41598-025-95899-7 (PMC12012204; doi:10.1038/s41598-025-95899-7)
Supplement: Supplementary file 1 — Supplementary Material 1 [file 41598_2025_95899_MOESM1_ESM.pdf]

Biosynthesis of Silver Nanoparticles by *Talaromyces funiculosus* for Therapeutic Applications and Safety Evaluation

Bahig A. El deeb[1,2*], Gerges G. Faheem[1] & Mahmoud S. Bakhit[1]

[1]Department of Botany and Microbiology, Faculty of Science, Sohag University, Sohag, 82524, Egypt

[2]Higher Technological Institute of Applied Health Science in Sohag, Ministry of Higher Education, Cairo, Egypt

*email: beldeep@science.sohag.edu.eg, bahig1978@gmail.com

Supplementary

Western blot analysis

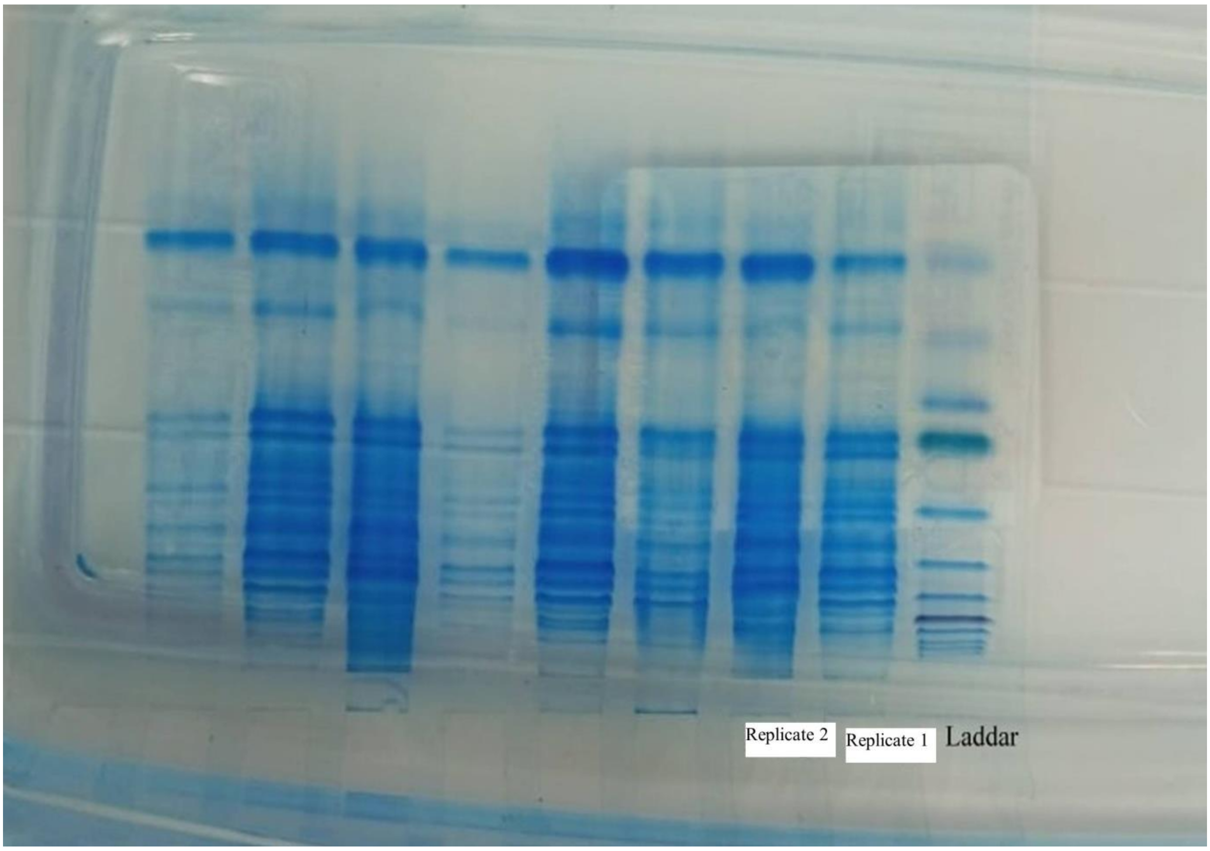

Supplemental Figure S1. Full SDS-PAGE gel image for Western blot data in Figure 12b and Figure 12c.

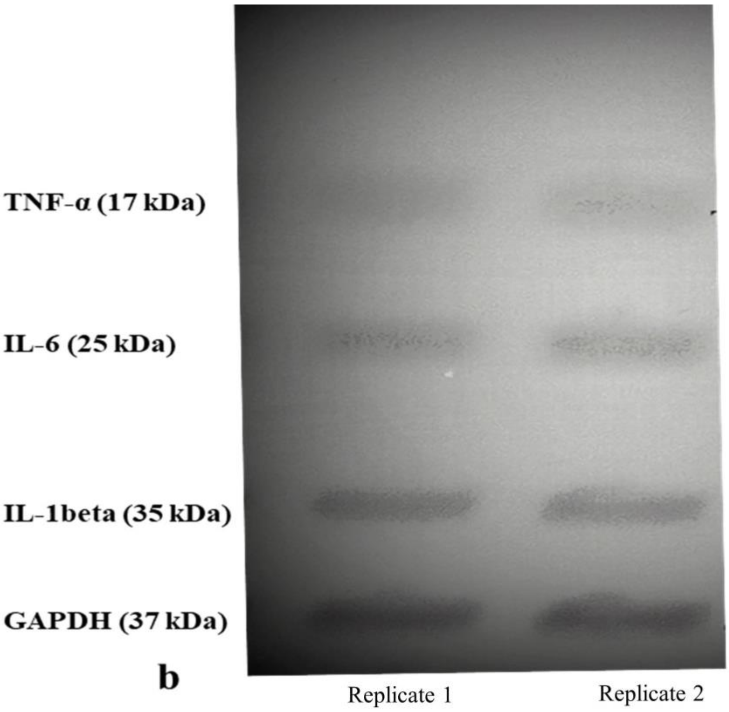

Figure 12b. Western blot image showing the protein expression of inflammatory cytokines (TNF-α, IL-6, and IL-1β).

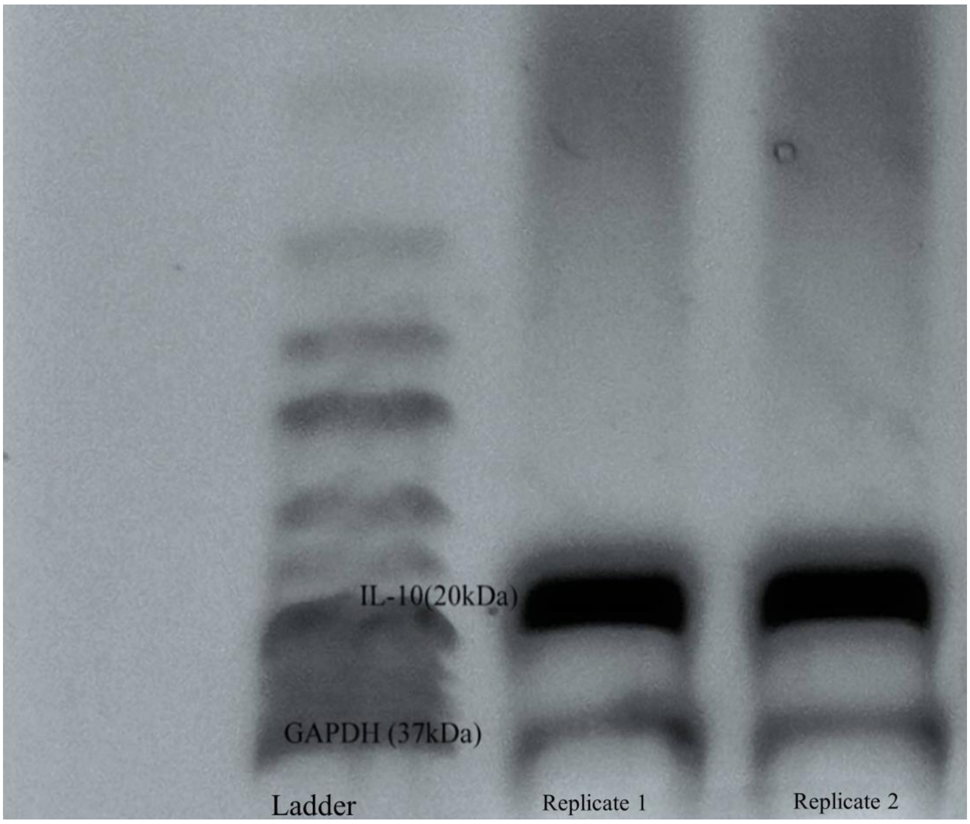

Figure 12c. Western blot image showing the protein expression of anti-inflammatory cytokine (IL-10).
